# The Kinetochore Protein Kis1/Eic1/Mis19 Ensures the Integrity of Mitotic Spindles through Maintenance of Kinetochore Factors Mis6/CENP-I and CENP-A

**DOI:** 10.1371/journal.pone.0111905

**Published:** 2014-11-06

**Authors:** Hayato Hirai, Kunio Arai, Ryo Kariyazono, Masayuki Yamamoto, Masamitsu Sato

**Affiliations:** 1 Laboratory of Cytoskeletal Logistics, Department of Life Science and Medical Bioscience, Graduate School of Advanced Science and Technology, Waseda University, TWIns, Tokyo, Japan; 2 Department of Biophysics and Biochemistry, Graduate School of Science, University of Tokyo, Tokyo, Japan; 3 Laboratory of Cell Responses, National Institute for Basic Biology, Aichi, Japan; 4 PRESTO, Japan Science and Technology Agency, Saitama, Japan; Virginia Tech, United States of America

## Abstract

Microtubules play multiple roles in a wide range of cellular phenomena, including cell polarity establishment and chromosome segregation. A number of microtubule regulators have been identified, including microtubule-associated proteins and kinases, and knowledge of these factors has contributed to our molecular understanding of microtubule regulation of each relevant cellular process. The known regulators, however, are insufficient to explain how those processes are linked to one another, underscoring the need to identify additional regulators. To find such novel mechanisms and microtubule regulators, we performed a screen that combined genetics and microscopy for fission yeast mutants defective in microtubule organization. We isolated approximately 900 mutants showing defects in either microtubule organization or the nuclear envelope, and these mutants were classified into 12 categories. We particularly focused on one mutant, *kis1*, which displayed spindle defects in early mitosis. The *kis1* mutant frequently failed to assemble a normal bipolar spindle. The responsible gene encoded a kinetochore protein, Mis19 (also known as Eic1), which localized to the interface of kinetochores and spindle poles. We also found that the inner kinetochore proteins Mis6/CENP-I and Cnp1/CENP-A were delocalized from kinetochores in the *kis1* cells and that kinetochore-microtubule attachment was defective. Another mutant, *mis6*, also displayed similar spindle defects. We conclude that Kis1 is required for inner kinetochore organization, through which Kis1 ensures kinetochore-microtubule attachment and spindle integrity. Thus, we propose an unexpected relationship between inner kinetochore organization and spindle integrity.

## Introduction

Microtubules are cylindrical polymers found in all eukaryotic cells and are involved in a number of cellular processes during the cell cycle [1], [2]. In interphase, cytoplasmic microtubules are arranged in a mesh-like array in animal cells or in a cylindrical array along the longitudinal axis in fission yeast. Cytoplasmic microtubules function to transport biomolecules within cells, and in this way the cytoplasmic array contributes to establishing cell polarity; for instance, the fission yeast cell-end marker protein, Tea1, is conveyed to cell tips via cytoplasmic microtubules [3].

In mitosis, centrosomes in animal cells and spindle pole bodies (SPBs) in yeast play central roles in the assembly of a microtubule-based bipolar spindle. Spindle microtubules capture and pull kinetochores formed on the centromeric DNA to segregate sister chromatids. The spindle elongates in anaphase using the antiparallel bundling of interpolar microtubules to further separate segregated chromosomes, and thus the spindle is required for faithful chromosome segregation and cell division. Therefore, all eukaryotic cells must alter microtubule organization between the cytoplasmic array and the spindle when entering or exiting mitosis.

Many studies in recent decades have identified a number of microtubule regulators. For example, the γ-tubulin complex is required for microtubule nucleation and controls the distribution and polarized growth of microtubules [4], [5]. Also, microtubule-associated proteins (MAPs) include motor proteins such as kinesin family proteins and dynein, factors involved in stabilization or destabilization of microtubules and factors that align or slide microtubule bundles [6], [7], [8]. The functional state of certain microtubule regulators varies during the cell cycle. In animal cells, the γ-tubulin complex is phosphorylated by Aurora A kinase for nucleation of microtubules to stabilize the spindle [9]. Aurora A also phosphorylates the conserved MAP TACC (transforming acidic coiled-coil protein) to recruit the TACC to the centrosomes [10], [11], [12]. Alp7, the fission yeast TACC ortholog, translocates from the cytoplasm to the nucleus upon entry into mitosis, which is critical for spindle formation [13]. A conserved microtubule bundling factor, Ase1/PRC1, as well as Klp9 (the fission yeast ortholog of kinesin-6) are dephosphorylated to bind to each other in anaphase, and the interaction is required for proper spindle elongation [14].

Although there is currently vast knowledge on this subject, how each aspect of microtubule functions and regulations are linked to each other at the molecular level remains largely unknown. More specifically, although much work has been done on microtubule functions and dynamics, the molecular mechanism(s) controlling microtubule regulation at specific cell cycle transitions remain largely unknown. For instance, it is not clear how microtubules are reorganized at mitotic entry and exit. In fission yeast, the reorganization of microtubules during the cell cycle can be summarized as follows (reviewed in [2], [15]). During interphase, a cytoplasmic microtubule array forms relatively uniformly along the cylinder formed by the yeast cell. Microtubule organizing centers (MTOCs) in interphase are thought to localize around the nucleus. Upon entry into mitosis, the cytoplasmic array of microtubules is disassembled and the mitotic spindle is formed in the nucleus. The main MTOC during mitosis is the SPB in *Schizosaccharomyces pombe*. Mitotic SPBs are embedded in the nuclear envelope, and in particular the nuclear side of SPBs nucleates spindle microtubules, whereas the cytoplasmic side forms an astral array of microtubules. Phosphorylation of the MAP complex TACC-TOG (Alp7–Alp14) by cyclin-dependent kinase 1 (Cdk1/Cdc2) contributes to the nuclear accumulation of the complex to assemble the spindle [16], [17]. In telophase, the mitotic spindle disassembles, and at roughly the same time a cytoplasmic MTOC in the equatorial ring at the cell center emerges (equatorial MTOC). Although these phenomena have been well characterized at the level of cell physiology, the molecular mechanisms remain largely unknown. We therefore speculated that uncharacterized factors may be critical for altering microtubule organization during the cell cycle.

To identify novel microtubule regulators, genome-wide RNA interference (RNAi) screens have been carried out in various experimental systems, including *Caenorhabditis elegans* [18], *Drosophila melanogaster* S2 cells [19], and human cell lines [20], [21]. Although these systematic screens have indeed identified new microtubule regulators, there may be practical concerns regarding their coverage. For instance, effective repression of gene expression using RNAi often needs fine-tuning with respect to the design of RNA oligomers, and this aspect might be insufficient in the case of large-scale RNAi screens. These issues may mask the real phenotype in the systematic knockdown screens. Moreover, RNAi experiments cannot be performed for unidentified genes that have not been annotated in databases.

Vizeacoumar et al. performed a high-content microscopy screen in combination with a systematic deletion library of the budding yeast *Saccharomyces cerevisiae* to explore spindle morphology [22]. Although a systematic deletion library of *S. pombe* is also available, here we chose a strategy of random mutagenesis instead of using this library for the following reason. Spindle regulators that contribute to spindle morphology might be essential for yeast viability, and thus deletion mutants of those factors would be expected to be inviable and therefore not included in the deletion library. To identify such essential factors, it is more appropriate to isolate conditional mutants with point mutations.

Methods for chemical mutagenesis have been firmly established in the long history of *S. pombe* studies, and a series of genetic screens have been performed to identify microtubule regulators [23], [24], [25]. To more efficiently find further novel microtubule regulators, here we combined a genetic screen with a visual screen so that we could isolate microtubule-deficient mutants directly through observation under the fluorescence microscope, without any bias from databases. Using such combination of forward genetic screen with microtubule visualization in living cells, we identified the protein, Kis1, which is required for spindle assembly in early mitosis and for inner kinetochore formation. We further show that a mutant of the inner kinetochore component Mis6 also displays spindle defects, suggesting a link between the inner kinetochore and spindle assembly.

## Results

### Designing a genetic-visual screen for mutants defective in microtubule organization

**Figure 1:**
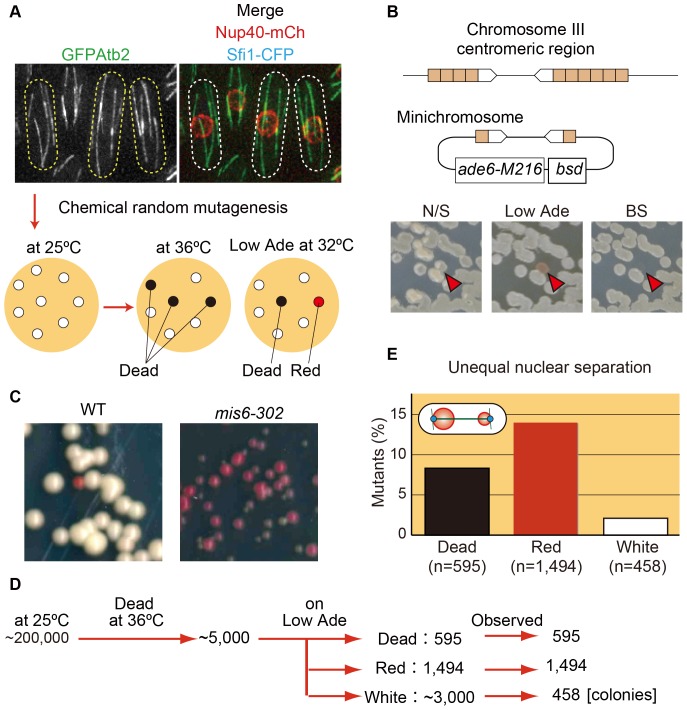
Scheme used for screening mutants having defects in microtubules or the nuclear envelope. (**A**) For the screen, a strain was prepared that had the microtubule marker (GFP-Atb2), the nuclear envelope marker (Nup40-mCherry), and the spindle pole body marker (Sfi1-CFP) as well as the minichromosome. After chemical random mutagenesis, colonies were grown at 25°C and replica-plated onto two plates, which were then incubated at 36°C or 32°C. The plate at 36°C was used to examine temperature sensitivity, and the plate at 32°C (Low Ade) was used to identify colonies showing possible minichromosome loss. Colonies showing both temperature sensitivity and possible minichromosome loss were chosen for subsequent visual screening. Cell shape is outlined by dotted lines. Bottom: Colonies are shown schematically as white, black, or red circles. (**B**) Top: Schematic drawings for the centromeric region of chromosome III and for the minichromosome, *CM3112sup3-5::ade6-M216-bsd*, used in this study. *CM3112sup3-5::ade6-M216-bsd* contains a part of the centromeric region and the selection markers indicated. Bottom: The strain was streaked on nonselective media (N/S) and replica-plated onto both Low Ade and blasticidin S (BS) plates. Arrowheads indicate a colony that lost the minichromosome. (**C**) Cells with minichromosomes in the wild-type (WT) and the *mis6-302* mutant background were streaked on Low Ade plates at 32°C for 5 (WT) or 8 days (*mis6-302*). (**D**) Summary of the screening results, showing the numbers of colonies obtained at each stage or category. (**E**) The population of mutants that showed unequal nuclear separation. Mutants were categorized based on whether they were dead or formed red or white colonies on Low Ade plates at 32°C.

We first designed a genetic screen to identify new factors that regulate microtubule organization during the cell cycle, particularly those involved in chromosome segregation. We previously established a methodology for construction and observation of “three-color” strains of *S. pombe* [26]. Specifically, cells are generated to express three proteins of interest fused to fluorescent proteins of different colors, so that the behavior of three cellular components can be simultaneously visualized in living cells. In general, strains express each fluorescent fusion protein at the level of the endogenous cellular protein to avoid possible artifacts caused by overexpression. For strains expressing green fluorescent protein (GFP)-tubulin (GFP-Atb2, the fusion protein of GFP and α2-tubulin), to visualize microtubule behavior we constructed the following two strains: the GFP-Atb2 strain expressing the fusion of GFP with Atb2 as the only copy of Atb2 [26], and the Z2-GFP-Atb2 strain expressing the fusion protein in addition to the endogenous Atb2 [27]. The latter strain appeared suitable for use in combination with severe mutation backgrounds that affect microtubule formation because the former GFP-Atb2 construct occasionally showed synthetic growth defects when combined with some mutations. Strain Z2-GFP-Atb2 has been used to elucidate previously unknown microtubule behaviors in various aspects of mitosis [26] and meiosis [27], [28], [29], [30]. We therefore prepared a three-colored strain expressing GFP-Atb2, Nup40-mCherry [31], and Sfi1-CFP (cyan fluorescent protein) [32] to monitor the behavior of microtubules, the nuclear envelope, and the SPB, respectively (**Figure 1A**).

The screening procedure consisted of three steps (**Figure 1A**). First, a minichromosome was introduced into the strain to detect chromosome mis-segregation in plate-based assays (see Materials and Methods for details); loss of the minichromosome causes red-colored adenine-auxotrophic mutant colonies on plates containing low concentrations of adenine (Low Ade) and no colony formation on plates with blasticidin S (**Figure 1B**). The *mis6-302* mutant, which causes severe minichromosome loss, frequently formed red-colored colonies (>95% of colonies) using the modified minichromosome, whereas only ∼5% of wild-type (WT) colonies were red (**Figure 1C**). This result validated the use of this CM3112-derived minichromosome for detection of minichromosome loss on plate-based assays.

As the three-colored strain with the minichromosome did not show growth defects at 25°C, 30°C, or 36°C (**Figure S1**), we used this strain as the WT strain for chemical mutagenesis. Cells were treated with nitrosoguanidine to introduce random mutations (**Figure 1A**). After plating cells onto rich media, we chose colonies that showed temperature-sensitive (ts) growth defects at 36°C and red (or red-sectored) color at 32°C, indicative of a possible minichromosome loss. Such candidate colonies were then subjected to microscopy at the restrictive temperature (36°C), and the organization of both microtubules and the nuclear envelope were monitored for defects.

We screened approximately 200,000 colonies on the initial plates, and ∼2.5% exhibited temperature sensitivity at 36°C (**Figure 1D**). The ts colonies were then classified into three categories based on growth and color on Low Ade plates at 32°C. Of the ts colonies, 12% showed almost no growth at 32°C (“dead”), 30% showed frequent red colonies, and 60% showed white colonies. We assessed nuclear envelope organization in ts mutants showing either dead or red colonies as well as in some of the white ts mutants for comparison to evaluate the reliability of the minichromosome-loss assay. Mutants in the red category showed more frequent unequal nuclear separation (14.1%, 210 mutants) than mutants in the white (2.2%, 10 mutants) or dead (8.6%, 51 mutants) categories (**Figure 1E**). These data demonstrated that the minichromosome-based screen efficiently identified mutants defective in chromosome segregation, although some other types of mutants, such as ones defective in DNA replication (minichromosome maintenance), were not excluded at this stage.

### Classification of mutants defective in organization of microtubules or the nuclear envelope

**Figure 2:**
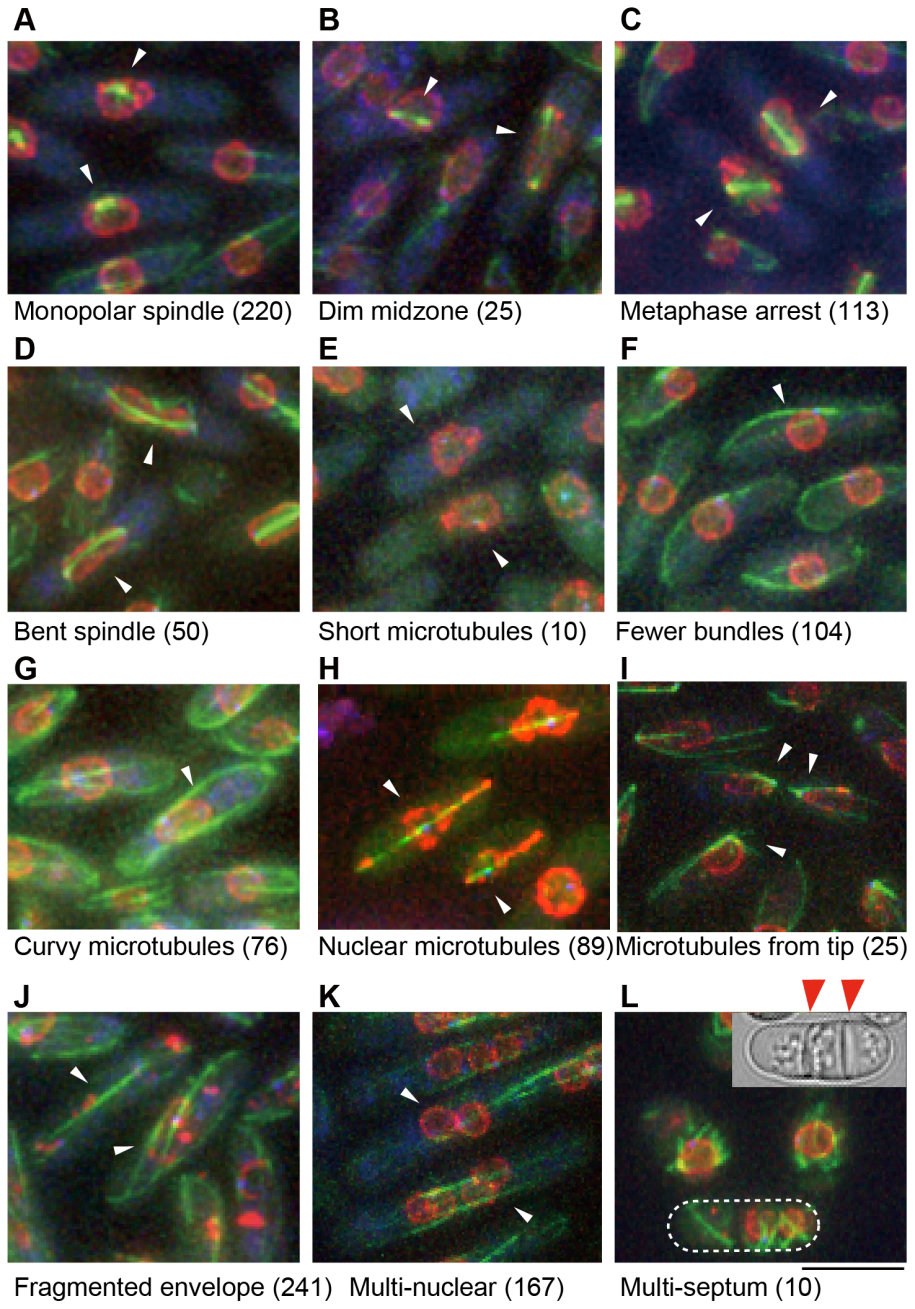
Isolated mutants showing defects in the organization of microtubules or of the nuclear envelope. GFP-Atb2 (green), Nup40-mCherry (red), and Sfi1-CFP (blue) of isolated mutants are shown. All mutants were observed after increasing the temperature to 36°C for 3–6 h. Cells displaying the typical phenotype are marked with white arrowheads. The numbers of mutants are also shown. Mutants were categorized according to their phenotype (A–L), and a brief explanation of each phenotype is as follows. (**A**) Monopolar spindle. (**B**) The middle region of the spindle showed a dim GFP signal. (**C**) Accumulation of cells within the metaphase spindle. (**D**) The spindle was bent in anaphase. (**E**) Extremely short microtubules. (**F**) The number of microtubule bundles was fewer than in wild-type cells. (**G**) Microtubules were elongated and curved at cell tips. (**H**) Microtubules formed in the nucleus during interphase. (**I**) Microtubules were tethered around the cell tip. (**J**) The nuclear envelope was fragmented. (**K**) Cells had more than one nucleus. (**L**) Multi-septated cells. A differential interference contrast image of the outlined cell is also shown. Red arrowheads indicate septa. Scale bar: 5 µm.

We then observed ∼2000 ts mutants showing dead or red colonies at 32°C. Of these, 42% displayed defects in interphase or mitosis and were classified into 12 categories according to phenotype (**Figures 2** **and S2**). Mutants showing multiple phenotypes were classified into each of the relevant categories **(Table S2)**.

Four categories included mutants showing defects in mitotic spindle morphology. For WT cells entering mitosis, the cytoplasmic microtubule array disappears and the spindle forms in the nucleus. The spindle is nucleated from two SPBs to yield a bipolar spindle. The spindle elongates in a stepwise manner: first the spindle elongates up to ∼2 µm (phase I), and the length is maintained for ∼5 min until chromosome segregation (phase II) [33]. After chromosome segregation, the spindle resumes elongating toward the cell tip (phase III) and cytoplasmic astral microtubules are formed at each SPB. In the first mutant phenotype category, the spindle formed from only one SPB, which is normally referred to as monopolar spindle (**Figures 2A** **and S2A**). This phenotype is common in a number of mutants for SPB components including Cdc31 [34], Sad1 [35], Cut12 [36], Cut11 [37], Pcp1 [38], Plo1 [39], and Cut7/Kinesin-5 [40]. In the second phenotype category, 25 mutants displayed spindles with a fragile midzone during early mitosis (**Figures 2B** **and S2B**), which is discussed in detail below. In the third category, 113 mutants frequently had short spindles. More than 20% of cells had 2 µm–long spindles (**Figures 2C** **and S2C**), suggesting that cells were arrested in metaphase. This phenotype is shared by mutants for components of the anaphase promoting complex [41], which plays an essential role in the metaphase-to-anaphase transition (reviewed in [42]). In the fourth category, 50 mutants showed normal spindle elongation in early mitosis, but the spindle became bent in the nucleus during anaphase (**Figures 2D** **and S2D**). A similar phenotype has been reported for the *skp1* mutant of the SCF (skp, cullin, F-box) ubiquitin ligase complex [30], [43]. This is probably due to entangled chromosomes that fail to resolve during mitosis and meiosis, which is caused abnormal tension in the elongating spindle [30], [43].

Five categories included mutants having microtubule defects during interphase. The WT interphase cells contained three to five bundles of microtubules, which generally run from the nuclear envelope toward the cell tips (reviewed in [44]). In the first category, however, 10 mutants exhibited almost no microtubule bundles (**Figures 2E** **and S2E**). This phenotype is reported in mutants of α- or β-tubulin [23], [45] and tubulin-folding cofactors [24], [46], [47], [48]. In the second category, 104 mutants tended to have fewer microtubule bundles (mostly one) than WT cells (**Figures 2F** **and S2F**). Mutants of γ-tubulin complex components and related proteins show these types of defects [49], [50], [51], [52], [53], [54], [55]. In the third category, 76 mutants showed abnormally long microtubules that curled at the cell ends (**Figures 2G** **and S2G**). Each mutant had more than one bundle, in contrast to the second category. This phenotype has been reported for *klp5*Δ and *klp6*Δ mutants; Klp5 and Klp6 are heterodimer kinesins that possibly disassemble microtubules [56], [57], [58]. In the fourth category, 89 mutants formed microtubules inside the nuclear envelope even during interphase (**Figures 2H** **, S2H and S3**). These cells were clearly in interphase because each cell had a single SPB. The nuclear shape was deformed into a lemon or skewered shape by elongation of microtubule protrusions abnormally formed in the nucleus. This phenotype has been reported in the *mto1*Δ *tip1*Δ double mutant, in which the spindle does not fully break down after mitosis, and nuclear microtubules persist to form a single bundle [59]. In the fifth category, 25 mutants had microtubules that appeared to be formed from sites near the cell tip (**Figures 2I** **and S2I**). This phenotype has been reported in the *rsp1-1* mutant [60]. In WT cells, the equatorial MTOC was assembled to tether microtubules at the site of cytokinesis in the mitotic telophase and disassembled after mitotic exit. In the *rsp1-1* mutant, however, the equatorial MTOC is not disassembled at mitotic exit, and microtubule bundles remain tethered during interphase of the next cell cycle [60].

Another category was designated for mutants displaying abnormalities in nuclear envelope morphology during interphase. WT cells uniformly showed the Nup40-mCherry signal on the surface of the nuclear envelope. In contrast, 241 mutants displayed a dotted pattern of Nup40-mCherry or did not show any particular localization of Nup40-mCherry in cells (**Figures 2J** **and S2J**), suggesting that the nuclear envelope was fragmented. Indeed, this phenotype is shared with mutants of nuclear pore complex components [61] or Pim1/RanGEF (the GTP/GDP exchange factor for the small GTPase, Ran), which orchestrates nucleocytoplasmic transport and nuclear envelope assembly [62].

The last two categories were for cytokinesis mutants. In the first category, 167 mutants often showed bi-, tetra-, or multi-nucleate cells (**Figures 2K** **and S2K**). This phenotype has been reported in cytokinesis mutants [63]. In the second category, 10 mutants contained cells with two or more septa (**Figures 2L** **and S2L**). This phenotype has been reported for mutants in which septation signaling (the SIN pathway; reviewed in [64], [65]) is ectopically activated.

### The *kis1-1* mutant displays defects in the central zone of the spindle

Among the vast number of mutants we categorized, we focused particularly on those that showed spindles with an abnormally dim GFP-Atb2 signal in the spindle midzone. During initial screening, we realized that some of those cells appeared to exhibit the phenotype when the spindle was still short, indicating the pre-anaphase state. Few mutants are known to show this phenotype before anaphase (as discussed later), and therefore we focused on mutants in this category; one such mutant was chosen and named *kis1-1* because of its function in kinetochore-microtubule interactions, as explained later.

**Figure 3:**
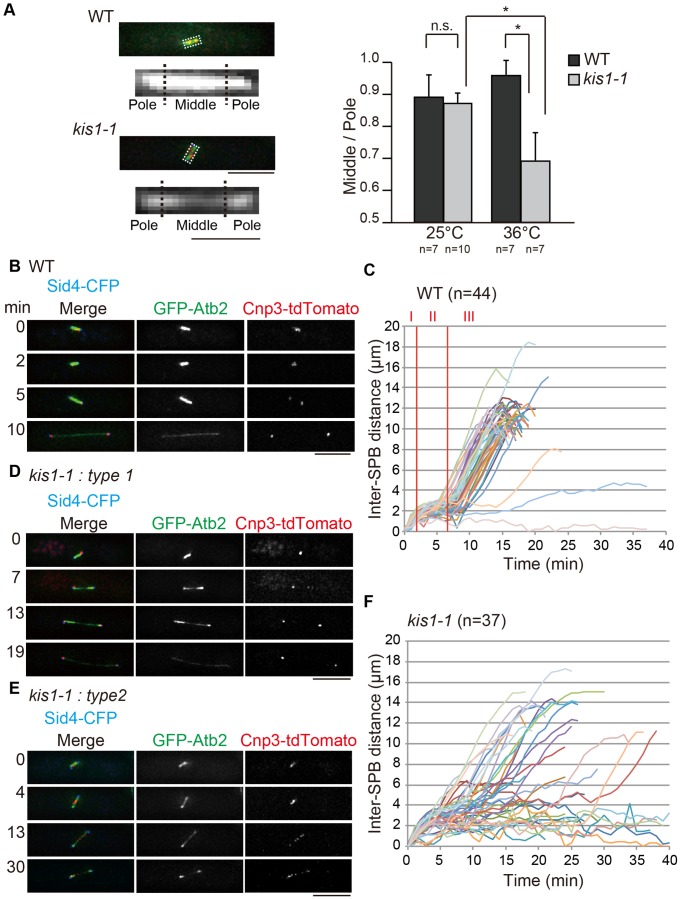
*kis1-1* has defects in spindle formation. Cells expressing GFP-Atb2 (marks microtubules), Cnp3-tdTomato (marks kinetochore), and Sid4-CFP (marks SPB) (**A,B,D,E**) and cells expressing Sfi1-GFP (**C,F**) in the indicated background were grown at 25°C, followed by a temperature shift to 36°C for 6–9 h. (**A**) Fluorescence intensity of the middle region of the metaphase spindle in *kis1-1* was dimmer than that in the wild-type (WT) strain. Left: The spindle was divided into three parts: the “middle” zone, the left “pole”, and right “pole.” The region outlined in each of the WT and *kis1-1* images, which shows the GFP-Atb2 signal, is magnified below each image. Right: The ratio of the “middle” GFP intensity to the “pole” intensity of spindles observed under the indicated conditions. *p<0.05 (Student's t-test); n.s., not significant (p>0.05). (**B–F**) Mitotic progression of WT and *kis1-1* cells. Kinetics of the inter-SPB distance in each of WT (**C**) and in *kis1-1* (**F**) are shown graphically. (**D,E**) *kis1-1* cells showed mainly two types of spindle defects: type 1, weak GFP-Atb2–staining midzone (D); and type 2, defects in spindle elongation (E). Scale bars: 5 µm except magnified images in A (1 µm).

First, to confirm that the midzone indeed had a dim GFP-Atb2 signal, each metaphase spindle was divided into the central zone and pole regions. The average GFP-Atb2 intensity was quantified for each region of WT and *kis1-1* cells, and their central-to-pole ratio was calculated (**Figure 3A**). The ratio in WT was ∼0.9 both at 26°C and 36°C. This may reflect the fact that most spindle microtubules in *S. pombe* are nucleated from SPBs [66]. In sharp contrast, the ratio in the *kis1-1* mutant dropped to ∼0.7

We have commonly observed an intense GFP-Atb2 signal in WT spindles (**Figure 3B** and **Movie S1**). The spindle starts to assemble when the two SPBs separate (**Figure 3B**), reaching ∼2 µm in length (this period of SPB separation is called phase I [67]) (**Figure 3C**). After 5∼10 min, the spindle resumes lengthening, and this corresponds to anaphase spindle elongation (phase III) (**Figure 3C**).

The *kis1-1* mutant frequently displayed two types of spindle defects. First, as mentioned above, spindles in prometaphase and metaphase showed a faint GFP-Atb2 signal (type 1; **Figures 3D** **and S4**) compared with WT spindles at the same stage, suggesting that microtubule nucleation or stabilization is defective in these cells. It was possible for most of these cells to elongate the spindle in anaphase, but the GFP-Atb2 signal in the midzone remained weak. Other common defects observed in *kis1-1* were spindle collapse or no elongation after reaching ∼2 µm (type 2; **Figure 3E** and **Movie S2**). Among 37 *kis1-1* cells, 32% showed this phenotype, and the cells failed to elongate the spindle in anaphase during the 20-min observation period (**Figure 3F**).

### *kis1* encodes an essential protein

**Figure 4:**
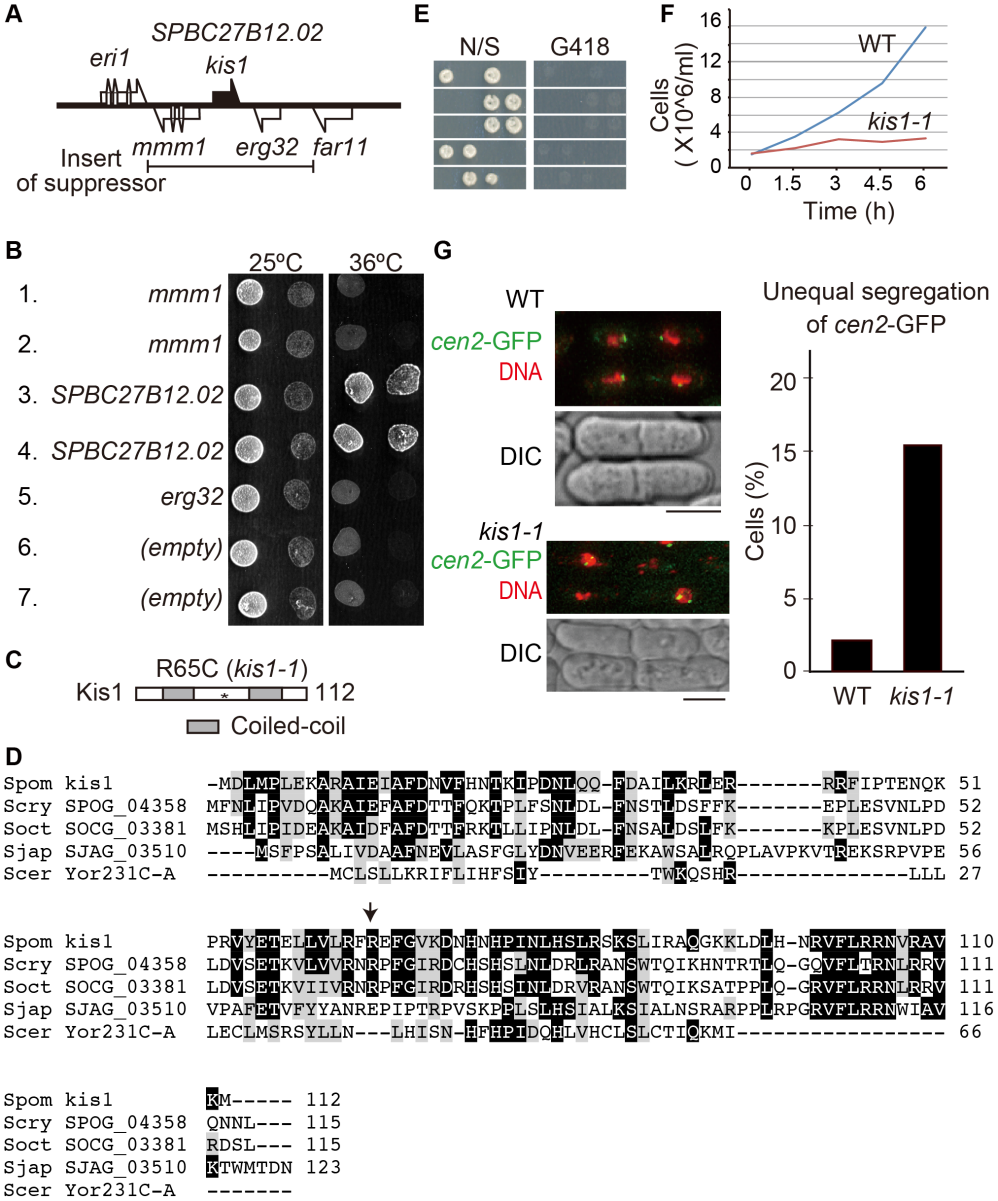
*kis1* encodes an essential protein. (**A, B**) Identification of *kis1*. (**A**) A multicopy suppressor plasmid of *kis1-1* contained an insert with the indicated region of chromosome II. (**B**) Ten-fold serial dilution assays on SD medium without leucine at 25 or 36°C. The *kis1-1* mutants harbor plasmids which express indicated genes (*mmm1*, *SPBC27B12.02* and *erg32*) or empty vector (empty). Plasmids in 1, 3, and 6 were derived from pREP1, whereas plasmids in 2, 4, 5, and 7 were derived from pREP41. The promoter in pREP1 is stronger than in pREP41. (**C**) Domain structure of Kis1. The asterisk indicates the mutation site of the *kis1-1* mutant (R65C). Gray boxes indicate predicted coiled-coil regions. (**D**) Alignment of Kis1/Mis19 with predicted orthologs in yeast. Identical residues are boxed in black. Similar residues are shaded in gray. Spom, *Schizosaccharomyces pombe*; Scry, *S. cryophilus*; Soct, *S. octosporus*; Sjap, *S. japonicus*; Scer, *Saccharomyces cerevisiae*. The arrow indicates the mutation site of *kis1-1*. (**E, F**) Kis1/Mis19 is essential for growth. (**E**) Diploid cells heterozygous for *kis1* (*kis1^+^/kis1::kan*) were sporulated and individual spores in each ascus were dissected on the nonselective medium (N/S). Two viable and two non-viable segregation patterns were obtained. Viable colonies were sensitive to kanamycin (G418) without exception, meaning that those are colonies of *kis1^+^* haploid cells. (**F**) Growth of WT and *kis1-1* cells at 36°C. (**G**) *kis1-1* displays unequal chromosome segregation. Left: Centromeres of chromosome II were visualized with GFP (*cen2*-GFP: green), DAPI (red), and differential interference contrast (DIC). Cells were grown at 36°C for 6 h. Right: Population of cells with unequal segregation of *cen2*-GFP. *n*>200.

To identify the gene responsible for the phenotype of *kis1-1*, we introduced the *S. pombe* genomic DNA library into *kis1-1* cells to search for clones that suppress the temperature sensitivity, and we isolated a DNA clone containing coding sequences of chromosome 2, i.e., *mmm1*, *SPBC27B12.02*, and *erg32* (**Figure 4A**). Subsequent complementation assays were performed with DNA clones containing one of the three coding sequences. *SPBC27B12.02* was the only gene of the three to complement the temperature sensitivity (**Figure 4B**). This gene was recently identified in two other studies [68], [69], in which it was named *mis19* and *eic1*. According to the *S. pombe* gene database PomBase [70], the registered gene name is *mis19*, and thus hereafter we refer to it as *kis1*/*mis19*.

PomBase indicated that the Kis1/Mis19 protein consists of 112 amino acid residues. According to the algorithm Phyre2, the protein contains two putative coiled-coiled regions [71] (**Figure 4C**). The *kis1-1* mutant gene has a single missense mutation (corresponding to substitution of arginine 65 to cysteine), which lies between the coiled-coil regions (**Figure 4C**). Although no homologous proteins were curated in PomBase, closely related yeast species had orthologs, e.g., SPOG_04358 of *Schizosaccharomyces cryophilus*, SOCG_03381 of *Schizosaccharomyces octosporus*, and SJAG_03510 of *Schizosaccharomyces japonicus* (**Figure 4D**), as noted previously [68], [69]. We also realized that the budding yeast *S. cerevisiae* appears to have a homologous protein (YOR231C-A), although it is a putative protein and sequence similarity is seen only in limited regions (**Figure 4D**). No putative orthologs were found in the databases for worm, fly, and mouse. A possible reason is that the size of Kis1/Mis19 orthologous genes might be so small that they were not yet annotated in the databases. Kis1/Mis19 has substantial sequence similarity with a part of an uncharacterized cytoplasmic protein in plants (*Arabidopsis* AT5G23520.1, 435 residues; data not shown) and with an isoform of the human kyphoscoliosis peptidase isoform X3 (XP_006713675, 681 residues; data not shown), which may be involved in muscle function [72].

In light of these results, we concluded that *mis19* ^+^ is the gene responsible for the *kis1-1* phenotype. *kis1/mis19* ^+^ was essential for growth, as indicated by a tetrad dissection assay (**Figure 4E**, also shown in [68]) and a growth curve assay (**Figure 4F**). Consistently, a systematic knockout screen by Bioneer indicated that complete deletion of the gene was lethal [73]. Mis19 is essential for normal chromosome segregation, as assessed by staining with DAPI (4′,6-diamidino-2-phenylindole) [68], [69]. Visualization of the centromeres of chromosome 2 using GFP (the *cen2*-GFP system [74]) revealed that ∼15% of *kis1-1* cells displayed missegregation of the GFP signal (**Figure 4G**), confirming that Kis1/Mis19 plays an important role in equal segregation of chromosomes in mitosis.

### Kis1/Mis19 localizes to the SPB-kinetochore interface during interphase

**Figure 5:**
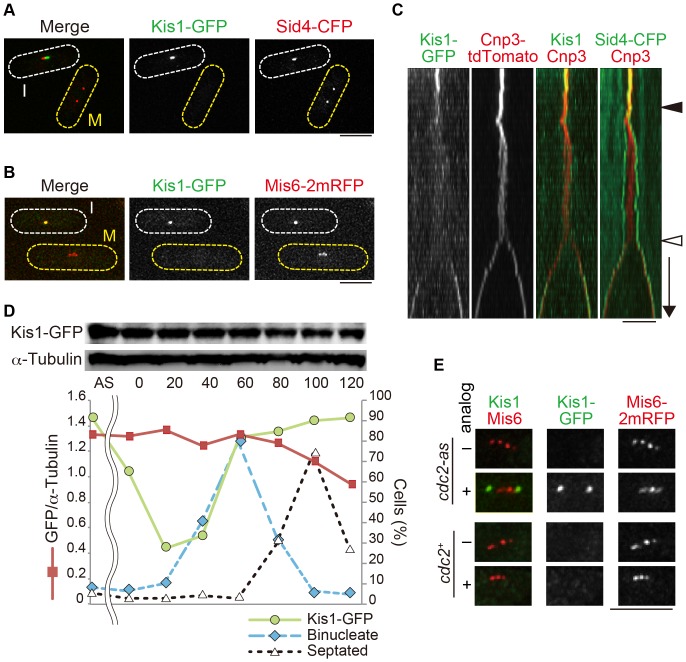
Kis1/Mis19 localizes to the kinetochore-SPB interface in interphase. (**A, B**) Kis1-GFP was visualized with Sid4-CFP (marks SPB; A) and Mis6-2mRFP (marks kinetochore; B). I: interphase cells; M: mitotic cells. Cell shapes are outlined. (**C**) Kymograph of Kis1-GFP and Cnp3-tdTomato in a cell filmed from G2 phase to anaphase. Kis1/Mis19 dispersed at the onset of SPB separation (filled arrowhead) and reappeared after chromosome segregation (open arrowhead). The length of the downward arrow corresponds to 2 min. (**D**) The amount of Kis1-GFP during the cell cycle. The *cdc25-22* mutant was used to synchronize the cell cycle at G2. Cells were shifted to 36°C for 4 h and then back to 25°C. Samples were then taken every 20 min (0–120 min). A sample for asynchronous cells (AS) was taken before the temperature shift. Top: Immunoblotting was performed with anti-GFP and anti-α-tubulin (control). Bottom: Kis1-GFP level was normalized with that of α-tubulin (red). Populations of cells with Kis1-GFP dots (green), binucleate cells (blue), and septated cells (black) are also shown. (**E**) Kis1-GFP was visualized with Sid4-CFP and Mis6-2mRFP in the *cdc2-as* (analog-sensitive) mutant. The ATP analog 1NM-PP1 was added to cells to inhibit the Cdc2 kinase activity, and images were acquired after 5 min after drug addition (analog, +). A mock-treated cell (analog, −) and *cdc2^+^* cells are also shown. Cells were cultured at 25°C except in (D). Scale bars, 5 µm.

We next investigated how Kis1/Mis19 regulates spindle microtubules. We tagged the endogenous *kis1/mis19* ^+^ on a chromosome with the GFP gene to express the Kis1-GFP fusion gene at the endogenous level. The GFP-tagged strain did not exhibit growth defects at 25, 30, or 36°C (**Figure S5**). During interphase, Kis1-GFP exhibited a single dot per cell that colocalized with the SPB marker Sid4-CFP [75] (**Figure 5A**). Kinetochores cluster around SPBs in interphase, whereas they dissociate from SPBs at mitotic onset [76]; thus, colocalization of Kis1-GFP and Sid4-CFP in interphase would indicate that Kis1/Mis19 may function at the SPB, kinetochore, or at their interface. To clarify this point, Kis1-GFP in mitotic cells was observed along with a kinetochore marker, Mis6-2mRFP (Mis6 tagged with two tandem copies of monomeric RFP) (**Figure 5B**). We noticed, however, that mitotic cells did not show any localization of Kis1-GFP (**Figure 5B**). Live-cell imaging confirmed that the Kis1-GFP dot at the SPB-kinetochore interface dispersed at mitotic onset, just before separation of SPBs and concomitant release of kinetochores from SPBs (**Figure 5C**). A similar observation was made in two recent reports (Mis19-GFP and Eic1-GFP) [68], [69]. Loss of Kis1-GFP localization in early mitosis was not due to degradation of the protein because lysates of cells that had been synchronized and released from the G2/M transition using the *cdc25-22* mutation [77] contained a constant amount of the Kis1-GFP during a 2-h period (**Figure 5D**).

Dispersion of Kis1-GFP from the SPB-kinetochore interface occurred only during early mitosis, as its localization recovered in anaphase. The transient exclusion of Kis1-GFP during early mitosis indicated that the dispersion may depend on mitotic kinases (Cdk1/Cdc2, Polo1, or Aurora). To investigate if the Kis1-GFP localization was regulated by Cdc2, we utilized the *cdc2-as* mutant, in which the kinase activity of Cdc2 could be inhibited by addition of the ATP analog, 1NM-PP1 [78], [79]. Kis1-GFP behaved normally in *cdc2-as* cells in the absence of 1NM-PP1, as in WT cells. When 1NM-PP1 was added, however, Kis1-GFP reappeared as dots at the SPB within 5 min (**Figure 5E**), thereby demonstrating that Kis1-GFP localization is indeed regulated by Cdc2 in mitosis.

### Mis6/CENP-I is displaced from the kinetochore in *kis1-1*

**Figure 6:**
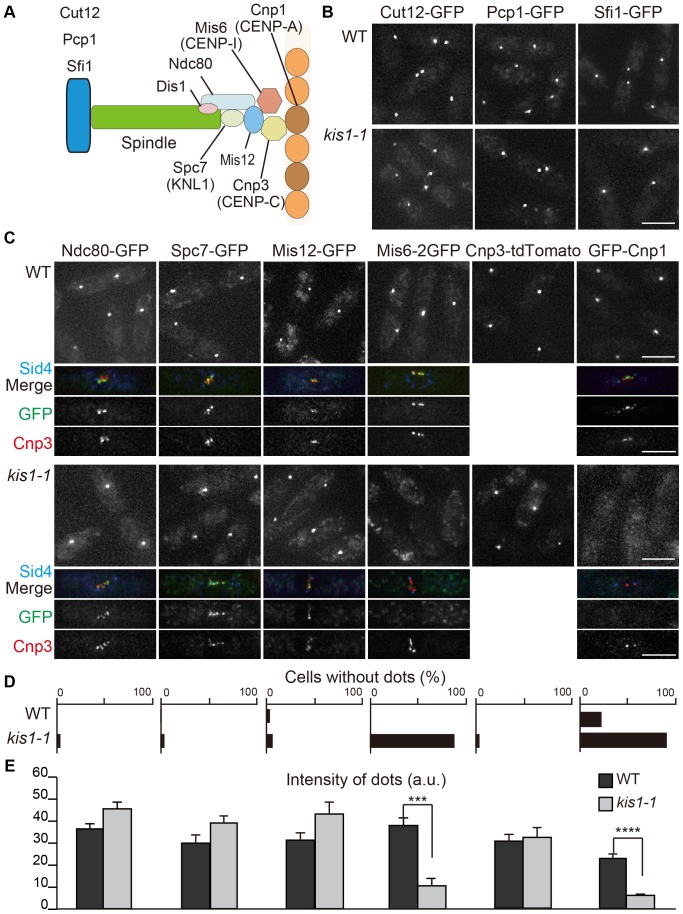
Localization of SPB and kinetochore proteins in *kis1-1*. (**A**) A diagram for SPB and kinetochore proteins examined in this study. (**B**) Localization of GFP-tagged SPB proteins (Cut12, Pcp1, and Sfi1) in WT and *kis1-1* cells at 36°C (6 h). (**C**) Localization of representative kinetochore proteins shown in (A) in WT and *kis1-1* cells. A typical mitotic cell for each GFP-tagged factor is exemplified below with Sid4-CFP and Cnp3-tdTomato. (**D**) Percentages of interphase cells without GFP or tdTomato localization at kinetochores in WT and *kis1-1* are shown for each kinetochore factor (*n*>50). (**E**) GFP or tdTomato fluorescence intensity at kinetochores in WT (black) or *kis1-1* (gray) cells. Cells were grown at 36°C for 6 h. *n*≥10. ***p<10^−4^; ****p<10^−6^ (Student's t-test). Scale bars, 5 µm.

The localization of Kis1-GFP at the kinetochore-SPB interface led us to postulate that Kis1/Mis19 might play a role in mitotic spindle assembly. *kis1-1* is a loss-of-function mutant because the mutant protein Kis1-1-GFP did not localize at the kinetochore-SPB interface at 36°C (**Figure S6**). The Eic1-1 mutant protein constructed by Subramanian et al. also showed reduced association with centromeres, albeit partially [69]. We considered that SPB structure might be affected in *kis1-1* cells and therefore examined the localization of representative SPB proteins (Cut12, Pcp1 and Sfi1; **Figure 6A**). No significant abnormalities were observed, however, within our limits of detection (**Figure 6B**).

**Figure 7:**
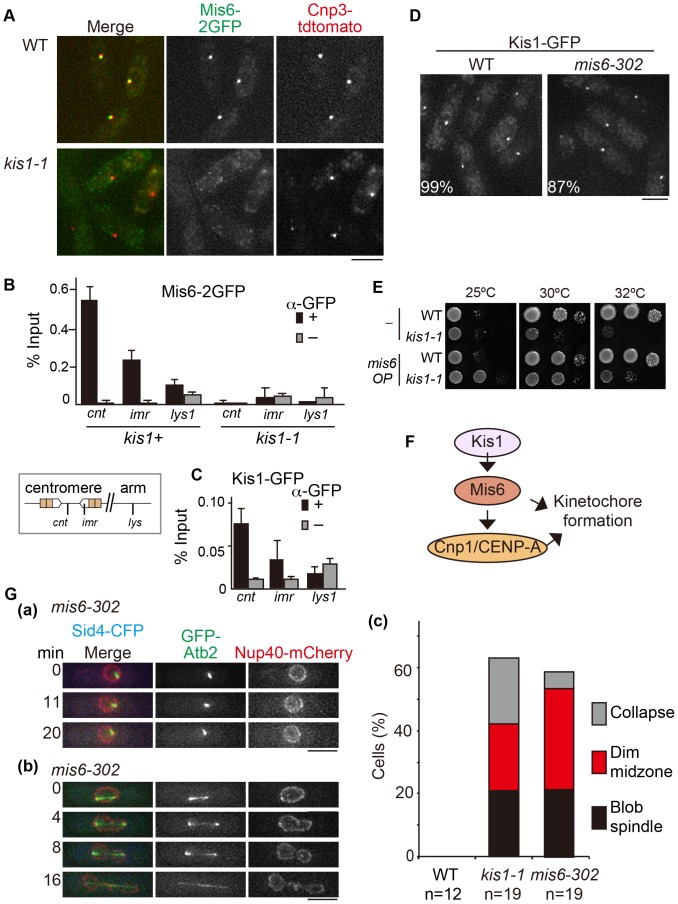
Kis1/Mis19 regulates Mis6 for spindle integrity. (**A**) Cells coexpressing Mis6-2GFP and Cnp3-tdTomato were imaged. (**B,C**) ChIP assays. Percentages of DNA precipitates of the centromeric region (*cnt* and *imr*) and the arm region (*lys1*) were measured as scaled to total input DNA using quantitative PCR. Black bars (+), immunoprecipitation with anti-GFP; gray bars (−), mock-treated negative control. Error bars indicate standard deviation (*n* = 3 reactions) (**B**) ChIP of Mis6-2GFP in WT or in *kis1-1* cells grown at 36°C for 6 h. (**C**) ChIP of Kis1-GFP. (**D**) Localization of Kis1-GFP in WT and *mis6-302* cells grown at 36°C for 6 h. Percentages indicate the proportion of interphase cells in which Kis1-GFP colocalized with both Cnp3-tdTomato and Sid4-CFP. (**E**) Ten-fold serial dilution assay. WT or *kis1-1* cells expressing either GFP (−) or Mis6-GFP (*mis6*) were grown on EMM plates at 25, 30, or 32°C. (**F**) A proposed model for localization dependency of Kis1/Mis19, Mis6 and Cnp1/CENP-A at kinetochores. (**G**) (a,b) Spindle phenotypes of *mis6-302* cells. Time-lapse imaging of GFP-Atb2, Nup40-mCherry, and Sid4-CFP in *mis6-302* was done at 36°C (6–9 h). (a) Blob spindles; (b) spindles with the dim midzone; (c) frequencies of spindle defects in WT, *kis1-1*, and *mis6-302* cells. Scale bars, 5 µm.

We next investigated whether Kis1/Mis19 may be involved in kinetochore organization rather than SPB functions. We thus examined the localization of kinetochore factors and chose representative components from distinct kinetochore subcomplexes. Those included three members of the KMN network, namely Spc7/Spc105/KNL-1 from the Knl1 subcomplex [80], Mis12/Mtw1 from the Mis12 subcomplex [81], and Ndc80 from the Ndc80-Nuf2 subcomplex [80], as well as two members of the constitutive centromere-associated network (CCAN), namely Mis6/CENP-I [82] and Cnp3/CENP-C [83], and the histone H3 variant Cnp1/CENP-A [83] (**Figure 6A**). By analyzing GFP-tagged versions of these proteins in both the WT and *kis1-1* background, we found that Mis6 and Cnp1, but none of the other kinetochore proteins analyzed, were displaced from the kinetochore in both interphase and mitotic cells at 36°C (**Figure 6C, D**). This was determined both visually and by quantification of fluorescence intensity (**Figure 6C, E**). A significant amount of Mis6-2GFP also appeared displaced at the permissive temperature, 25°C (**Figure S7**). Displacement of Mis6 from kinetochores at 36°C was confirmed by simultaneous visualization of Mis6-2GFP and Cnp3-tdTomato (**Figure 7A**). The level of Mis6 and Cnp1 was normal in *kis1-1* cells at 36°C (**Figure S8)**. Hayashi et al. found similar results regarding displacement of Mis6 and Cnp1 [68].

The requirement of Kis1/Mis19 for Mis6 localization was then confirmed by chromatin immunoprecipitation (ChIP) of Mis6-2GFP (**Figure 7B**). Reduced association of Mis6 to the central core region of centromeres was also shown by Subramanian et al. [69]. Furthermore, a ChIP assay with Kis1-GFP demonstrated that Kis1/Mis19 accumulated at centromeric DNA (**Figure 7C**), as also shown recently [68], [69]. Taken together, these results suggest that Kis1/Mis19 associates with centromeres to facilitate Mis6 localization.

Localization of CENP-A depends on Mis6 in fission yeast [83], suggesting that a dysfunctional Kis1-1 mutant caused delocalization of Mis6 from kinetochores during mitosis, which led to a loss of CENP-A. By contrast, Kis1-GFP localized normally in the *mis6-302* ts mutant (**Figure 7D**) [68]. Also, association of Eic1-GFP with centromeres is not altered in the *mis6-302* mutant [69]. These results indicate that Mis6 is not required for Kis1/Mis19/Eic1 localization.

To this point, our data suggested that *kis1-1* has at least two defects: disorganization of the spindle midzone and loss of Mis6 and CENP-A, although SPB factors appeared intact. We thus wondered if these two defects are linked to each other or completely independent and wanted to clarify whether the defect in the spindle midzone in *kis1-1* originated from an abnormal loss of Mis6 from kinetochores. Hence, we first overexpressed Mis6 in *kis1-1*, which partially suppressed the temperature sensitivity of *kis1-1* at the semi-restrictive temperature (32°C, **Figure 7E**). We concluded that the main function of Kis1/Mis19 is to recruit Mis6 to kinetochores, which in turn function to maintain CENP-A–containing nucleosomes at centromeres (**Figure 7F**; [83]).

### The *mis6-302* mutant displays spindle defects similar to those observed in *kis1-1*

To determine whether the loss of Mis6 seen in *kis1-1* was also responsible for spindle defects, we performed live-cell imaging of *mis6-302* cells and found that they frequently displayed spindle defects at the restrictive temperature (36°C). *mis6-302* mainly showed two types of spindle defects in mitosis: an extremely short and non-elongating spindle (termed “blob” spindle) (**Figure 7G** **a**), and a spindle with a dim GFP-Atb2 signal in the midzone (“dim midzone”) (**Figure 7G** **b**). The frequency of the blob spindle (∼20%) in *mis6-302* was similar to that in *kis1-1* (**Figure 7G** **c**). Frequencies of dim midzone and spindle collapse were also similar to those in *kis1-1*, demonstrating that both *kis1* and *mis6* mutants were defective in spindle integrity at a similar level. Thus, we concluded that defects in a part of the inner kinetochore organization seen in *mis6-302* and *kis1-1* affected the spindle integrity and that Kis1/Mis19 is required to ensure spindle integrity through organization of the inner kinetochore.

### Kis1/Mis19 genetically and physically interacts with the Mis16-Mis18 complex

**Figure 8:**
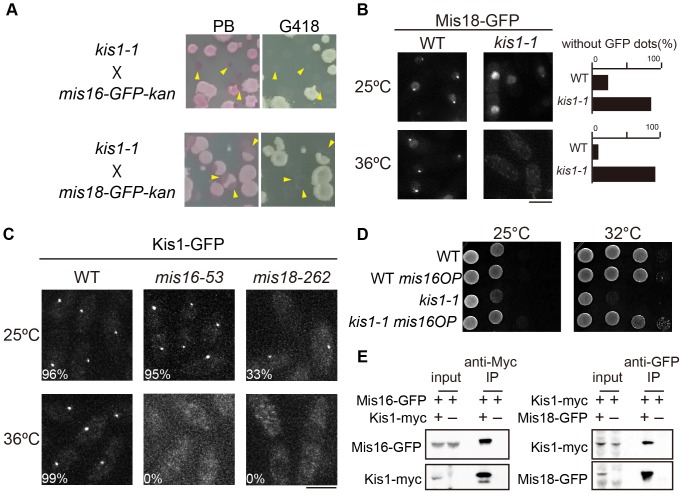
Genetic and physical interaction between Kis1/Mis19 and the Mis16-Mis18 complex. (**A**) Genetic crossing of *kis1-1* and *the mis16-GFP-kan* or *mis18-GFP-kan* mutant was performed, and spores were germinated on YE5S plates at 25°C. Colonies were then replica-plated onto YE5S plates with phloxine B (PB) or with kanamycin (G418) and incubated at 36°C or 25°C, respectively. The temperature-sensitive colonies (arrowheads) were sensitive to G418 without exception, indicating that the double mutant was inviable. *mis16* and *mis18* were found on the chromosome III, whereas *kis1/mis19* was on chromosome II, excluding the possibility of genetic linkage between *kis1/mis19* and *mis16/mis18*. (**B**) WT or *kis1-1* cells expressing Mis18-GFP from plasmids were observed after growth at 25 or 36°C for 6 h. Percentages of cells without Mis18-GFP dots are shown (n≥100). (**C**) Localization of Kis1-GFP in WT, *mis16-53*, or *mis18-262* cells grown at 25 or 36°C for 6 h. Frequencies are also given (n≥100). Scale bars, 5 µm. (**D**) Overexpression of Mis16-GFP suppressed the temperature sensitivity of *kis1-1*. Cells harboring plasmid pREP1-mis16-GFP (*mis16 OP*) were spotted on EMM plates and incubated at 25 or 32°C. Cells harboring pREP1-GFP were spotted as controls. (**E**) Coimmunoprecipitation (IP) of Kis1 and the Mis16–Mis18 complex. Cell extracts (input) were prepared from cells expressing (+) or not expressing (−) the indicated proteins. Kis1-myc (left) and Mis18-GFP (right) were immunoprecipitated with anti-Myc or anti-GFP, respectively. IP samples as well as 2.5% of the input were analyzed.

As the requirement of Kis1/Mis19 for kinetochore localization of Mis6 and Cnp1 indicated functional similarity with Mis16-Mis18 [84], a genetic interaction between *kis1* and *mis16* (or *mis18*) was tested. As shown in **Figure 8A**, GFP-tagged strains of *mis16* and *mis18* could not form colonies in the *kis1-1* background, suggesting that GFP-tagging of Mis16 and Mis18 compromised their functions and that both *mis16-GFP-kan* and *mis18-GFP-kan* were synthetically lethal with *kis1-1*. Similar synthetic growth defects were recently shown by Subramanian et al [69]. Because of the lethality, we introduced multicopy plasmids expressing Mis18-GFP in addition to endogenously expressed Mis18 to monitor whether Mis18-GFP localized appropriately in the absence of Kis1/Mis19. **Figure 8B** demonstrates that Mis18-GFP failed to localize to kinetochores in *kis1-1* cells at 36°C. Kis1-GFP also failed to localize to kinetochores in both *mis16-53* and *mis18-262* mutant cells at the restrictive temperature (36°C) (**Figure 8C**). Similar results were also obtained by Hayashi et al. (microscopy) and Subramanian et al. (ChIP) [68], [69]. Moreover, Mis16 overexpression suppressed the temperature sensitivity of *kis1-1* (**Figure 8D**), and immunoprecipitation assays using *S. pombe* extracts revealed a physical interaction between Kis1/Mis19 and Mis16–Mis18 (**Figure 8E**). The two aforementioned groups very recently isolated the Mis19/Eic1 protein via biochemical purification of the Mis16-containing kinetochore subcomplex [68], [69]. From these results, we conclude that Kis1/Mis19 is a component of the Mis16–Mis18 complex.

### Kis1/Mis19 ensures kinetochore-microtubule attachment

How does Kis1/Mis19 organize spindle microtubules through kinetochores? We assumed that kinetochores without functional CENP-A or Mis6 in *kis1-1* may be defective in attachment to spindle microtubules, and the lack of kinetochore-microtubule interaction may cause the destabilization of microtubules.

**Figure 9:**
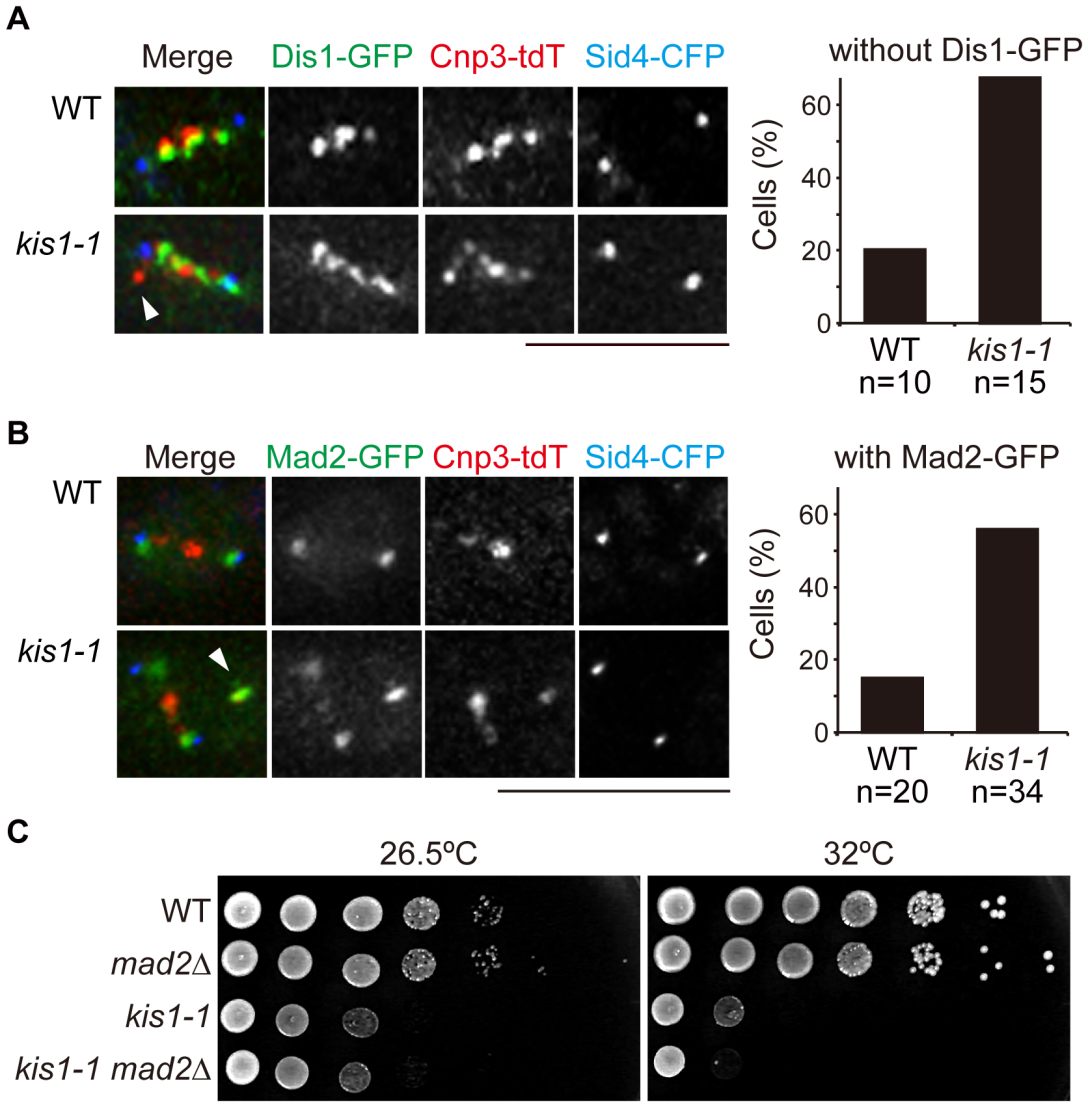
Spindle organization in *kis1-1*. (**A**) Dis1 was not loaded onto unattached kinetochores in *kis1-1*. Left: Dis1-GFP was visualized together with Cnp3-tdTomato and Sid4-CFP in WT and *kis1-1* cells after growth at 36°C for 6–9 h. Arrowheads indicate a kinetochore without Dis1-GFP. Right: Percentages of mitotic cells having one or more kinetochores without Dis1-GFP. (**B**) Mad2-GFP localized to unattached kinetochores in *kis1-1*. Left: Mad2-GFP in WT and *kis1-1* cells was observed with Sid4-CFP and Cnp3-tdTomato after growth at 36°C for 6–9 h. Arrowheads indicate kinetochores with Mad2-GFP. Right: Percentages of metaphase cells bearing Mad2-GFP dots at kinetochores. *n*: The number of cells analyzed. Scale bar, 5 µm. (**C**) Ten-fold serial dilutions of the indicated strains spotted on YE5S at 25 or 32°C.

To assess if kinetochore-microtubule attachment was defective in *kis1-1*, we analyzed the subcellular localization of the microtubule-associated protein Dis1 [85], an ortholog of human ch-TOG and *Xenopus laevis* XMAP215, which accelerates microtubule growth (reviewed in [86], [87]). In *S. pombe*, Dis1 localizes to the outer surface of kinetochores in metaphase by binding to Ndc80 [88], [89], [90]. Given that the *dis1*Δ mutant exhibits a weak-staining spindle midzone as in *kis1-1* [90], Kis1/Mis19 may be required for Dis1 localization to kinetochores to stabilize the spindle. When we examined Dis1-GFP more closely, we found that only 20% of WT metaphase cells (*n* = 10 cells) lacked Dis1 at kinetochores, indicating that kinetochore-microtubule association had been established (**Figure 9A**). In contrast, 66.6% of *kis1-1* cells in metaphase (*n* = 15) exhibited kinetochores without Dis1-GFP, and those kinetochores were often located away from the spindle (**Figure 9A**). Consistently, Dis1 displays reduced association to centromeres in the absence of microtubules [88]. This strongly suggested that the kinetochore-microtubule attachment was indeed defective in *kis1-1*.

In general, unattached kinetochores are monitored by spindle-assembly checkpoint proteins including Mad2 to prevent unequal chromosome segregation (reviewed in [91], [92]). In WT metaphase cells, Mad2-GFP localized to SPBs, indicating that the checkpoint was not active at that time because kinetochore-microtubule attachment was established [93] (**Figure 9B**). In *kis1-1*, Mad2-GFP frequently localized to unattached kinetochores (**Figure 9B**), a hallmark of checkpoint activation. The temperature sensitivity of *kis1-1* at the semi-restrictive temperature (32°C) deteriorated when the spindle assembly checkpoint was disabled by deletion of *mad2* (the *kis1-1 mad2*Δ double mutant, **Figure 9C**). Tracking of the inter-SPB distance indicated that the kinetics of spindle elongation in the *kis1-1 mad2*Δ double mutant was ameliorated compared with the *kis1-1* single mutant (**Figure S9**). Overall, these observations demonstrate that kinetochore-microtubule attachment is defective in *kis1-1*, and this, in turn, activates the spindle assembly checkpoint and causes spindle elongation defects.

## Discussion

Our screen for ‘*kis*’ mutants aimed to isolate factors that are essential for assembly of the spindle midzone, particularly in early mitosis. The *kis1* mutant isolated in the screen showed defects in the organization of the inner kinetochore in addition to spindle midzone defects. Mis6/CENP-I and Cnp1/CENP-A of the inner kinetochore proteins were particularly delocalized in the *kis1* mutant.

### Kis1/Mis19 is a member of the Mis16-Mis18 complex

Very recently, Kis1 (also named as Mis19 or Eic1) was independently isolated by two groups using biochemical assays that searched for new components of the Mis16–Mis18 complex [68], [69]. Their biochemical and genetic analyses demonstrated that Kis1/Mis19/Eic1 is a member of the Mis16–Mis18 complex. In contrast, we used a genetic-visual screen to isolate the same gene as a factor regulating spindle morphology. We demonstrated that kinetochore-microtubule attachment was defective in *kis1-1*. Our observations regarding microtubule organization and functions were not described in the other two reports. The data reported here together with the data reported by these other studies collectively provide a comprehensive perspective of the mechanism underlying the relationship between kinetochores and microtubules: Kis1/Mis19 and Mis16–Mis18 are required for localization of inner kinetochore components, including Mis6/CENP-I and CENP-A. These factors not only construct kinetochores on centromeres but also stabilize the spindle. This was evidenced by the observed lack of spindle midzone stabilization in *kis1-1*, even though the outer kinetochore factors, which serve to dock microtubules, remained intact. The spindle defects seen in *kis1-1* and *mis6-302* cells were very similar, but the genetic suppression of *kis1-1* by Mis6 overexpression was partial. Mis6 overexpression may not be sufficient to target it to kinetochores because Mis18, which promotes Mis6 localization, was also delocalized in *kis1-1*. Alternatively, Kis1/Mis19 might play other roles in addition to recruitment of Mis6 to kinetochores.

### Kis1/Mis19 is required for kinetochore maintenance during interphase

Kis1/Mis19 is expected to function at the kinetochore-SPB interface because the loss-of-function mutant Kis1-1 failed to localize properly at 36°C (see **Figure S6**). Nonetheless, in WT cells, Kis1/Mis19 disperses upon entry into mitosis, when kinetochores play indispensable roles for faithful chromosome segregation.

It is possible that the architecture of the inner kinetochore is constructed in interphase using SPBs as a docking site and using Kis1/Mis19 and Mis16–Mis18 as a scaffold. As cells enter mitosis, the scaffold may need to be dismantled to release kinetochores from the dock. It was very recently shown that, in human cells, artificial localization of Mis18α (a component of the Mis18 complex) to centromeres caused constitutive CENP-A loading during mitosis, which resulted in defects in chromosome alignment at metaphase [94]. This implies that Kis1/Mis19-Mis16-Mis18 needs to be removed for a similar reason.

The mechanism for the dispersion of Kis1/Mis19 and Mis16–Mis18 remains unclear, but our analysis demonstrates that the dispersion of Kis1/Mis19 from the SPB-kinetochore interface depends on Cdc2/Cdk1. When Cdc2 activity was artificially inhibited in mitosis, Kis1/Mis19 relocalized to SPBs rather than kinetochores (see Figure 5E), suggesting that Kis1/Mis19 preferentially localizes to SPBs, presumably to build the scaffold for kinetochore assembly for use in the next cell cycle. Substrate(s) of Cdk1 for regulation of Kis1/Mis19-Mis16-18 localization remains to be elucidated. In human and chicken DT40 cells, Cdk1 phosphorylates the Mis18-binding protein Mis18BP1 [94], [95], although fission yeast has no apparent orthologs of Mis18BP1. Kis1/Mis19 does not have consensus sites for phosphorylation by Cdk1, but both Mis16 and Mis18 have consensus sites, and one of the Mis16 sites has been reported as a phosphorylation site in a high-throughput phosphoproteome assay [96]. This raises the possibility that phosphorylation of Mis16–Mis18 by Cdc2 might disperse the complex that includes Kis1/Mis19 from the kinetochore-SPB interface upon entry into mitosis.

### Spindle assembly checkpoint is active in the *kis1* mutant

Our experiments showed that *kis1-1* displayed spindle defects, which activated the spindle assembly checkpoint, as indicated by Mad2-GFP accumulation at unattached kinetochores (**Figure 9B**). It has been reported, however, that the checkpoint is not activated in *mis6-302*, and therefore Mis6 serves as the platform on kinetochores for Mad2 localization [97]. In human HeLa cells, Mad2 localization to unattached kinetochores also depends on CENP-I/Mis6, although CENP-I-depleted cells only displayed a transient delay in the metaphase-to-anaphase transition [98]. Although *kis1-1* also lacked Mis6 at kinetochores, Mad2 kinetochore localization persisted. Our data suggest that Mad2 may bind to yet another platform factor in addition to Mis6 in fission yeast, although we cannot rule out the possibility that residual Mis6 localization in *kis1-1* might be sufficient for Mad2 localization.

### Inner kinetochore proteins regulate kinetochore-microtubule attachment

*mis6-302* also showed spindle defects (blob spindle and fragile midzone) similar to those of *kis1-1* (**Figure 7G**). Our results thus demonstrate that a lack of inner centromere proteins (Mis6/CENP-I and CENP-A) causes spindle defects. Importantly, outer kinetochore proteins serving as microtubule-binding sites remained intact in *kis1-1*, suggesting that inner centromere proteins play an essential function independent of their well-known role in the outer kinetochore architecture [99], [100], [101]. It remains to be elucidated how the kinetochore structure is damaged in *kis1-1*, but we speculate that the inner part of kinetochores may be loosened by lack of Mis6 and CENP-A. Kinetochores without Mis6 and CENP-A may still be able to interact with the lateral surface of microtubules because outer kinetochore components are intact, but the interaction may not be properly converted to end-on attachment. Therefore, the plus-end tracking MAP Dis1/TOG may not be supplied from microtubules to kinetochores, which results in unstable attachment as evidenced by unattached kinetochores without Dis1 (**Figure 9A**).

Our combinatory screen of genetics and microscopy thus identified a factor for microtubule regulation. Although it is universally accepted that the outer kinetochore plays an essential role in kinetochore-microtubule attachment, our study illuminates the importance of the inner kinetochore for attachment, which acts independently of outer kinetochore components. Gene identification and functional analyses of other ‘*kis*’ factors may identify additional factors involved in spindle organization, particularly those that have already been identified as kinetochore proteins.

## Materials and Methods

### Yeast genetics and strains

Table S1 lists the strains used in this study. Standard methods for yeast genetics and gene tagging were used [26], [102], [103], [104]. For visualization of GFP-tubulin, the gene for GFP-Atb2 (α2-tubulin), regulated by the *atb2* promoter and terminator, was integrated into a chromosome as an extra copy of endogenous *atb2* ^+^ (referred to as “Z2-GFP-Atb2”) [27]. Media used in the study were YE5S (a rich medium containing 0.5% yeast extract, 2% glucose, and 75 µg/ml adenine, histidine, leucine, uracil, and lysine), Low Ade (same composition as YE5S except for 7.5 µg/ml adenine), EMM and SD. For Figure 8B, 2 µg/ml phloxine B was added on YE5S.

To construct the strain used for the mutagenesis (KA2165; Table S1), we modified CM3112, a circular minichromosome of approximately 36 kb in size derived from *S. pombe* chromosome III [105]. The original minichromosome contains the *sup3-5* marker gene, and we replaced it with two tandemly aligned markers, *ade6-M216* and the blasticidin S resistance gene *bsd*, to avoid rescue of introduced nonsense mutations by the nonsense suppressor *sup3-5* [106]. The construct was made as follows: *ade6-M216* with the *ade6* promoter and terminator was amplified by PCR and then cloned into PacI-AscI sites of pCR2.1-TOPO in which *bsd* had already been inserted. The resultant plasmid pCR2.1-ade6-M216-bsd had tandem genes *ade6-M216* and *bsd* and was used as the template for the standard PCR-based gene-targeting method to delete *sup3-5*. Cells were grown in YE5S medium containing blasticidin S before mutagenesis, minichromosome loss assays (Figure 1B, C), or dilution assays (Figure S1) to select the cells harboring the minichromosome.

For visualization and protein studies of GFP-Cnp1 and for visualization of Mis18-GFP, genomic *cnp1* and *mis18* were amplified by PCR, and cloned into the plasmid pREP1-GFP to express the GFP-fusion protein under the *nmt1* promoter. Cells with the plasmids were cultured in the SD medium without leucine. To overexpress Mis6-GFP and Mis16-GFP, *mis6* and *mis16* were cloned into the plasmid pREP1-GFP, respectively. Cells containing the plasmids were cultured on EMM plates without leucine.

### Microscopy

Live-cell imaging was performed with the DeltaVision-SoftWoRx system (GE Healthcare) as described [26]. Briefly, cultured cells were transferred to a glass-bottom dish and were supplied with EMM for observation. Images were acquired through *z*-sectioning, deconvolution, and stacking using the ‘quick projection’ algorithm of the SoftWoRx software. Temperature conditions were as follows: in Figures 5A, B, C and E, and S7, and in the upper panel of 8B, 8C, S6 (labeled 25°C), cells were grown at 25°C. In Figures 1, 2 and S2, cells cultured in 96-well plates were grown at 25°C followed by a shift to 36°C for 3 h. Each mutant was observed sequentially under the microscope. The large-scale observation was terminated before the total incubation time reached 6 h at 36°C. Images taken during screening were, hence, taken at 3–6 h after the temperature shift. For other figures, cells were grown at 36°C for 6 h or 6–9 h (for time-lapse imaging).

To measure the pole-to-pole distance (Figures 3C, F and S9), 10-section images were acquired along the *z* axis at 0.4-µm intervals, deconvolved, and merged into a single projection. The center of a single Sfi1-GFP dot was considered as the SPB position, and the distance between two center points was defined as the pole-to-pole distance. Live-cell imaging started when the pole-to-pole distances were less than 1 µm (defined as time 0).

To measure the fluorescence intensity of kinetochore factors visualized with GFP or tdTomato, the mean intensity of fluorescence in 7×7 pixilated squares was measured, and the extracellular background fluorescence was subtracted. For GFP-tagged proteins, the position of kinetochores was defined by the Cnp3-tdTomato dot signal. For quantification of Cnp3-tdTomato localization, strains expressing Mis6-2GFP (KRY211, HH78) were used.

To inhibit Cdc2 kinase activity, a modified version of the analog-sensitive *cdc2* mutant (*cdc2-asM17*; [79]) expressing Kis1-GFP (KRY202) was used. Either dimethyl sulfoxide was added to a mock-treated sample (−analog) or 2 µM 1NM-PP1 (Calbiochem) was added to inhibit Cdc2 activity (+analog).

For Figure 4G, cells were fixed with 3.2% formaldehyde for 15 min, and DNA was stained with DAPI (Wako Pure Chemicals). Images were acquired with an Axioplan 2 fluorescence microscope (Zeiss) and SlideBook software (Leeds Precision).

For Figure 5D, Kis1-GFP was observed without cell fixation, whereas the nucleus and the septum were observed after fixation with 30% ethanol at 4°C followed by DAPI staining.

### Mutagenesis and colony selection

The strain KA2165 (Table S1) was mutagenized with 0.3 mg/ml nitrosoguanidine, as described [107]. Cells were then spread onto YE5S plates and incubated at 25°C. Colonies were replica-plated onto YE5S containing 2 µg/ml phloxine B (36°C) and onto Low Ade plates (32°C). Cells that had lost the minichromosome turned red on Low Ade plates because the *ade6-M210* mutant allele was no longer suppressed by the *ade6-M216* allele located on the minichromosome. Colonies that showed both ts at 36°C and a red color or no growth at 32°C were chosen in the initial screen. In total, ∼200,000 mutagenized colonies were screened.

### Identification of *kis1*

*kis1-1* was backcrossed to ensure the linkage between its temperature sensitivity and the spindle phenotype. *kis1-1* was transformed with pART genomic DNA library [108]. Transformants that grew on SD plates without leucine at 25°C were replica-plated onto YE5S containing phloxine B and then incubated at 34°C. Plasmids that suppressed the temperature sensitivity were isolated from grown colonies followed by sequencing. The DNA fragment on the plasmid contained three coding genes (see text for details). To identify which gene was responsible for suppression, each gene was PCR-amplified and cloned into pREP1 or pREP41 and used for the complementation assay.

### Protein studies

Proteins were extracted from cells using standard procedures as follows: for Figure 5D, cells were cultured in YE5S medium at 25°C for 18 h, and a portion of the culture was collected as an asynchronous sample. The rest of the culture was shifted to 36°C for 4 h and then shifted back to 25°C. At each time point after the temperature decrease (0–120 min), cells were washed with STOP buffer (150 mM NaCl, 50 mM NaF, 10 mM EDTA pH 8.0, 1 mM NaN_3_). For other figures, cells were cultured at 25°C, or cultured at 25°C followed by a temperature shift to 36°C for 6 h and washed with STOP buffer. After centrifugation, cell pellets were collected and frozen at −80°C.

As needed, frozen cell pellets were suspended in Lysis buffer (50 mM Tris-HCl pH 7.5, 1 mM EDTA, 150 mM NaCl, 0.05% NP-40, 10% glycerol, 1 mM dithiothreitol, 1 mM phenylmethanesulfonyl fluoride, Complete protease inhibitor cocktail (Roche)) and then lysed with glass beads in a FastPrep-24 beads shocker (MP Biomedicals, 25 s×2−3 times, power 5.5) at 4°C. After 2–3 rounds of centrifugation at 5800× *g* for 2 min at 4°C, the supernatants were collected (total cell extract). For immunoprecipitation, each cell extract was incubated with anti-c-Myc [9E10] (1∶600; Santa Cruz Biotechnology) or rabbit polyclonal anti-GFP (1∶600; Life Technologies) for 1.5 h at 4°C. Protein G–coupled DynaBeads (Life Technologies) were added and incubated for 1.5 h at 4°C. Beads were washed four times with Lysis buffer and suspended in Lysis buffer containing SDS sample buffer and boiled. The resulting samples were resolved on 4–12% gradient polyacrylamide gels (Bio-Rad) and transferred to nitrocellulose membranes using the iBlot Dry Blotting system (Invitrogen, Program P3). For immunoblotting, SNAP i.d. 2.0 (Millipore) was used. Each membranes was blocked with 0.1% skim milk in phosphate-buffered saline containing 0.1% Tween 20 and blotted with anti-c-Myc [9E10] (1∶1000), anti-GFP [7.1 and 13.1 mixture] (1∶1000; Roche), or anti-α-tubulin [B-5-1-2] (1∶2000; Sigma) as the primary antibody; horseradish peroxidase–conjugated anti-mouse (1∶2500; GE Healthcare) was used as the secondary antibody. For Figures 5D and S8, the band intensities in fixed-size boxes were quantified using ImageJ (http://imagej.nih.gov/ij/). The ratios of GFP-tagged protein to α-tubulin intensity were calculated (the intensity of an unload lane was subtracted as the background).

### ChIP

ChIP was carried out essentially following standard procedures [83], [109]. For Figure 7B, cells were incubated at 25°C in YE5S followed by a temperature shift to 36°C for 6 h and fixed with 1% formaldehyde. Cells were incubated at 36°C for 10 min and for 50 min on ice. For Figure 7C, cells were grown at 25°C and fixed with 1% formaldehyde at 25°C for 10 min and then on ice for 50 min.

Fixed cells were then washed twice with phosphate-buffered saline and once with Buffer I (50 mM Hepes/KOH pH 7.5, 140 mM NaCl, 1 mM EDTA, 1% Triton X-100, 0.1% sodium deoxycholate and Complete Protease Inhibitor (Roche)) at 4°C. Cell pellets were stored at −80°C and as needed were suspended in Buffer I and lysed with glass beads using the FastPrep-24 beads shocker (4×25 s; power setting  = 6.0). After removing the glass beads by centrifugation, the chromatin was sheared to 200–800 bp using the VP-050 sonicator (TAITEC, 15 rounds of 50 pulses (0.2 s each) at a 30% power output). The extracts were centrifuged at 17,800× *g* for 3 min, and the supernatants were taken and centrifuged likewise for another 15 min. The protein concentration of each extract was measured with the Bio-Rad Protein Assay kit and adjusted to 20 mg/ml with Buffer I. For each input sample, 50 µl of extract was used. For ChIP samples, 500 µl extract was incubated with or without anti-GFP Living Colors Full-length polyclonal antibody (1∶250; Clontech) for 1.5 h. Protein G–coupled DynaBeads (Life Technologies) were then added and incubated for 1.5 h. Beads were washed twice with Buffer I, once with Buffer I containing 0.5 M NaCl, once with Buffer II (10 mM Tris-HCl pH 8.0, 250 mM LiCl, 0.5% NP-40, 0.5% sodium deoxycholate) and twice with TE. For both input and ChIP samples, the composition of DNA fragments was assessed by quantitative PCR with Power SYBR Green using the StepOne Real Time PCR system (Applied Biosystems). Established primers were used for PCR as follows [109]: For the central core region of centromeres (cnt), 5′-ATCTCATTGCTATTTGGCGAC-3′ and 5′-GCGTTTCCTTCGGCGAAATGC-3′. For the inner repeat region (imr), 5′-CACATACCAAAAAGTCTGGC-3′ and 5′-GCTGAGGCTAAGTATCTGTT-3′. For an example of the arm region (the *lys1* gene locus), 5′-ATTTTCGCATCCAACGCTGC-3′ and 5′-ACAACTAAGGCTCTGGGCTT-3′.

## Supporting Information

Figure S1 **The parental strain of the screen does not exhibit growth defects.** The parental strain of the screen carries the minichromosome (minichromosome, +) and three fluorescent markers: GFP-Atb2, Sfi1-CFP, and Nup40 (three-colored marker, +). Serial dilutions of cells were spotted on YE5S plates and incubated at 25, 30, or 36°C. The strain without minichromosome (minichromosome, –) and the strain lacking all three fluorescent markers (three-colored marker, –) are also shown as controls.
(TIF)

Figure S2 **Representative cell chosen from each phenotype category.** Cells were observed after increasing the temperature to 36°C for 3–6 h. Images for three-colored markers, namely, GFP-Atb2 (green), Sfi1-CFP (blue), and Nup40-mCherry (red); corresponding merged images are shown in the top row. Categories in this figure are identical to those in Figure 2. **(A)** Monopolar spindle. **(B)** The middle region of the spindle had a dim GFP signal. **(C)** Accumulation of cells within the metaphase spindle. **(D)** The spindle was bent in anaphase. **(E)** Extremely short microtubules. **(F)** The number of microtubule bundles was fewer than in WT cells. **(G)** Microtubules were elongated and curved at cell tips. **(H)** Microtubules formed in the nucleus during interphase. **(I)** Microtubules were tethered around the cell tip. **(J)** The nuclear envelope was fragmented. **(K)** Cells showing more than one nucleus. **(L)** Multi-septated cells. Arrowheads indicate SPBs. Scale bar: 5 µm.
(TIF)

Figure S3 **Serial images along the** ***z*** **axis of a cell with nuclear microtubules during interphase.** The microtubule bundle is in the nuclear envelope. A cell in the category “Nuclear microtubules” (Figure 2H) was observed after increasing the temperature to 36°C for 4 h. Images were acquired at 0.4-µm intervals along the *z* axis. The stacked image and images of each section are shown for GFP-Atb2, Sfi1-CFP and Nup40-mCherry. Scale bar: 5 µm.
(TIF)

Figure S4 **Sequential images of the** ***kis1-1*** **cell of** **Figure 3D** **taken at 1-min intervals.**
(TIF)

Figure S5 **GFP-tagging of Kis1 does not affect cell proliferation.** Ten-fold serial dilution assays of cells expressing GFP-tagged Kis1 (*kis1-GFP*) or non-tagged Kis1 (WT) were spotted on YE5S plates and incubated at 25, 30, or 36°C.
(TIF)

Figure S6 **Kis1-1-GFP did not localize to kinetochores at 36°C.** Cells expressing GFP-tagged Kis1 (WT, A) or GFP-tagged Kis1-1 (B) were visualized together with Sid4-CFP and Cnp3-tdTomato at 25 or 36°C (6 h). Left: Images for representative cells. Right: GFP fluorescence intensity at kinetochores in interphase cells observed under the indicated conditions. ***p<10^−4^ (Student's t-test); n.s., not significant (p>0.05), *n*≥10.
(TIF)

Figure S7 **Localization of kinetochore proteins in WT or** ***kis1-1*** **at 25°C.** Cells were cultured at 25°C (the permissive temperature) and imaged. These data serve as controls for Figure 6C–E, in which cells were cultured at 36°C (the restrictive temperature). **(A)** Localization of the indicated kinetochore proteins observed in WT or *kis1-1* cells. For mitotic cells, Sid4-CFP and Cnp3-tdTomato are also shown below. **(B)** Frequency of interphase cells without GFP localization at kinetochores for each kinetochore factor. *n*>50. **(C)** GFP or tdTomato fluorescence intensity of each kinetochore factor in WT (black) or *kis1-1* (gray) cells. *n*≥10. ***p<10^−4^; ****p<10^−6^ (Student's t-test). Scale bars, 5 µm.
(TIF)

Figure S8 **Amount of Mis6-2GFP and GFP-Cnp1 in WT or in** ***kis1-1*** **.** Cell extracts were prepared from WT or *kis1-1* cells expressing Mis6-2GFP **(A)** or GFP-Cnp1 **(B)** cultured at 25 or 36°C (6 h). Immunoblotting was performed with anti-GFP and anti-α-tubulin. Cell extracts without Mis6-2GFP and GFP-Cnp1 are also shown as negative controls (–). A ratio value is shown for the Mis6-2GFP band intensity (A) or GFP-Cnp1 (B) intensity compared with that for α-tubulin.
(TIF)

Figure S9 **Kinetics of the inter-SPB distance in the** ***kis1-1 mad2Δ*** **double mutant.** The *kis1-1 mad2Δ* double mutant cells expressing Sfi1-GFP (SPB) were grown at 25°C, followed by a temperature shift to 36°C for 6–9 h. Images were acquired every minute, and the inter-SPB distance was measured for each time point. *n* = 8.
(TIF)

Table S1 **Fission yeast strains used in this study.**
(PDF)

Table S2 **The number of mutants classified into one or two categories.** In the screen, each mutant was classified into 1 or more of 12 categories according to phenotype. Category names are the same as shown in Figure 2, labeled as (A) to (L). Some mutants exhibited two distinct phenotypes and thus were assigned to both relevant categories. For example, 17 mutants belong to both (A) and (C) categories. The number of mutants classified into only one (“in one”) or in two categories (“in two”) is shown for each cell. Total numbers are also shown as a reference.
(PDF)

Movie S1 **Mitotic progression in WT.** Movie showing the mitotic progression of a WT cell expressing GFP-Atb2 (green) and Sid4-CFP (red). The cell was grown at 36°C for 3–6 h, and images were acquired every minute for 14 min. Scale bar, 5 µm.
(MOV)

Movie S2 **Spindle collapse phenotype in** ***kis1-1*** **.** A *kis1-1* mutant cell expressing GFP-Atb2 (green) and Sid4-CFP (red) was grown at 36°C for 3–6 h, and images were acquired every minute for 24 min. This movie corresponds to data presented in Figure 3E. Scale bar, 5 µm.
(MOV)
