# Supplementary figures and images for: The Kinetochore Protein Kis1/Eic1/Mis19 Ensures the Integrity of Mitotic Spindles through Maintenance of Kinetochore Factors Mis6/CENP-I and CENP-A
Source: PLoS One. 2014 Nov 6;9(11):e111905. doi: 10.1371/journal.pone.0111905 (PMC4222959; doi:10.1371/journal.pone.0111905)

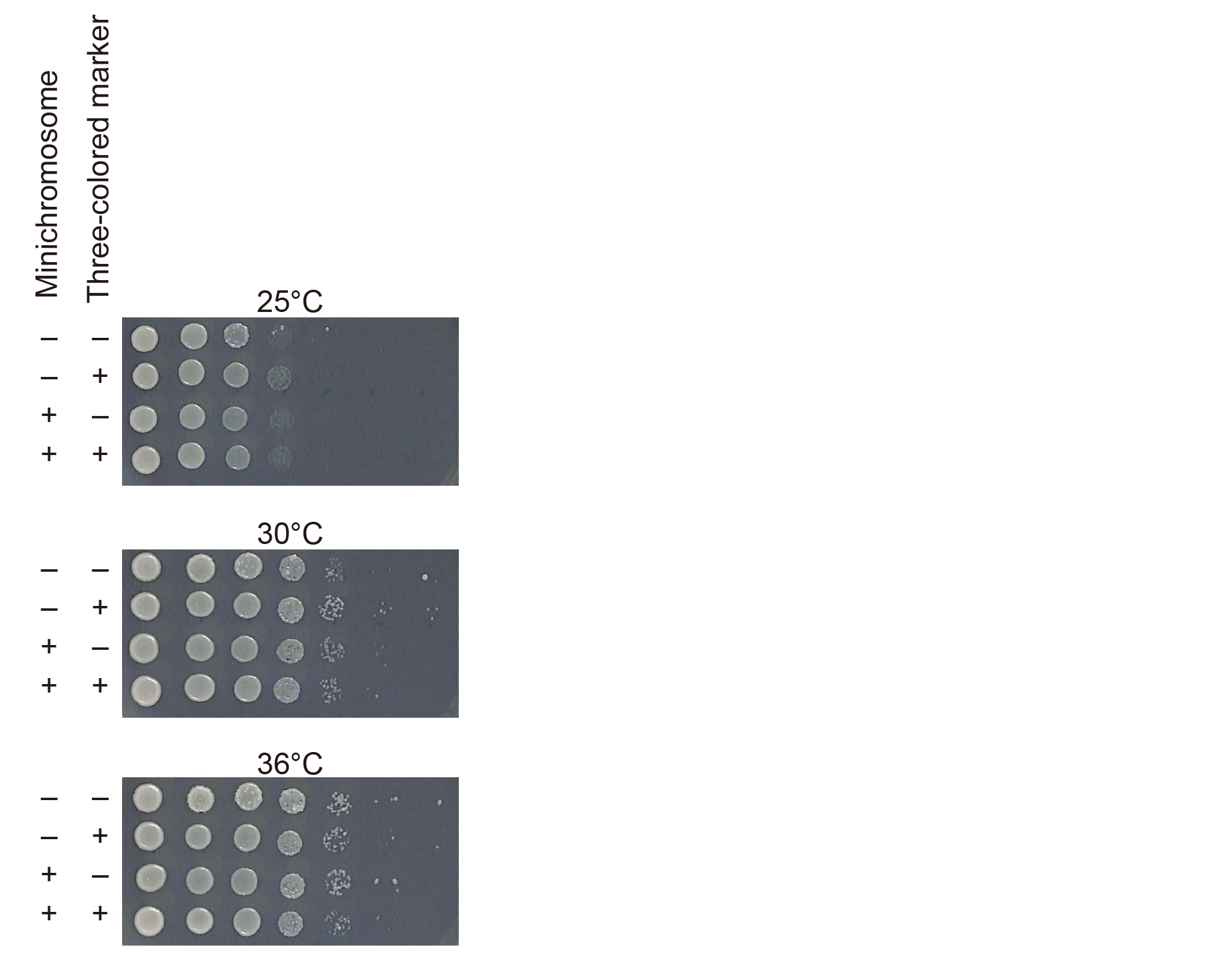

Supplement: Figure S1 — The parental strain of the screen does not exhibit growth defects. The parental strain of the screen carries the minichromosome (minichromosome, +) and three fluorescent markers: GFP-Atb2, Sfi1-CFP, and Nup40 (three-colored marker, +). Serial dilutions of cells were spotted on YE5S plates and incubated at 25, 30, or 36°C. The strain without minichromosome (minichromosome, –) and the strain lacking all three fluorescent markers (three-colored marker, –) are also shown as controls. (TIF) [file pone.0111905.s001.tif]

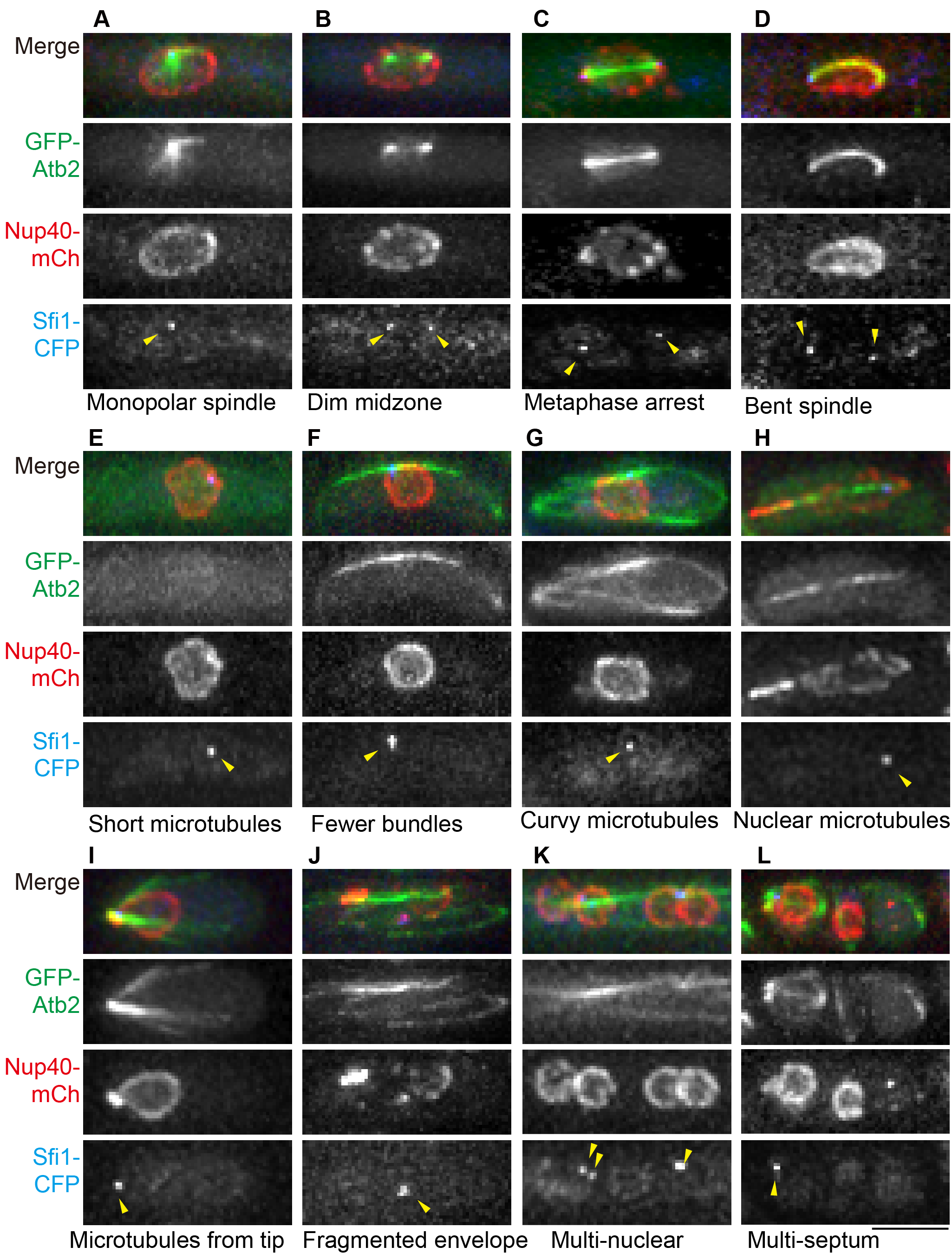

Supplement: Figure S2 — Representative cell chosen from each phenotype category. Cells were observed after increasing the temperature to 36°C for 3–6 h. Images for three-colored markers, namely, GFP-Atb2 (green), Sfi1-CFP (blue), and Nup40-mCherry (red); corresponding merged images are shown in the top row. Categories in this figure are identical to those in Figure 2. (A) Monopolar spindle. (B) The middle region of the spindle had a dim GFP signal. (C) Accumulation of cells within the metaphase spindle. (D) The spindle was bent in anaphase. (E) Extremely short microtubules. (F) The number of microtubule bundles was fewer than in WT cells. (G) Microtubules were elongated and curved at cell tips. (H) Microtubules formed in the nucleus during interphase. (I) Microtubules were tethered around the cell tip. (J) The nuclear envelope was fragmented. (K) Cells showing more than one nucleus. (L) Multi-septated cells. Arrowheads indicate SPBs. Scale bar: 5 µm. (TIF) [file pone.0111905.s002.tif]

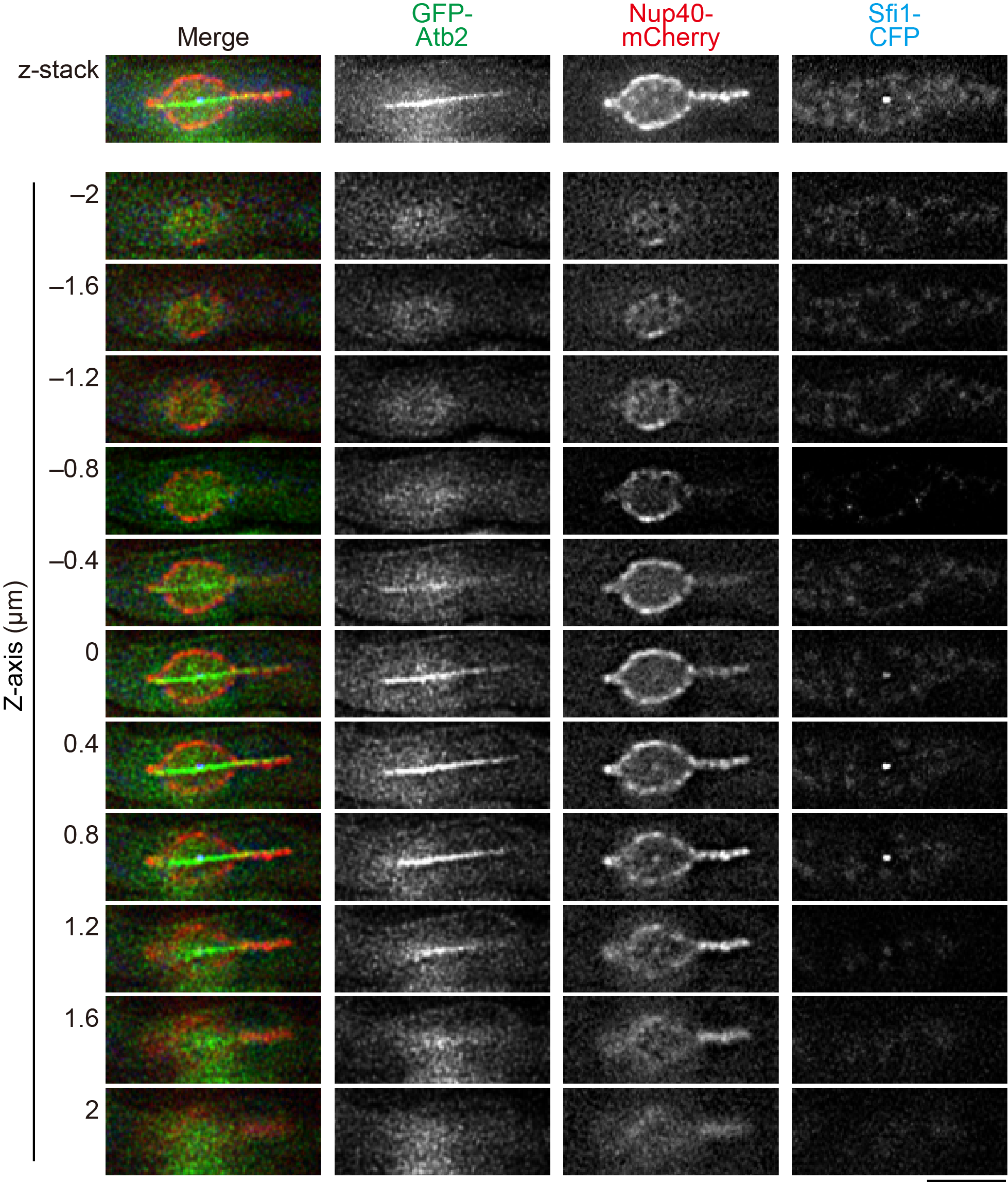

Supplement: Figure S3 — Serial images along the z axis of a cell with nuclear microtubules during interphase. The microtubule bundle is in the nuclear envelope. A cell in the category “Nuclear microtubules” (Figure 2H) was observed after increasing the temperature to 36°C for 4 h. Images were acquired at 0.4-µm intervals along the z axis. The stacked image and images of each section are shown for GFP-Atb2, Sfi1-CFP and Nup40-mCherry. Scale bar: 5 µm. (TIF) [file pone.0111905.s003.tif]

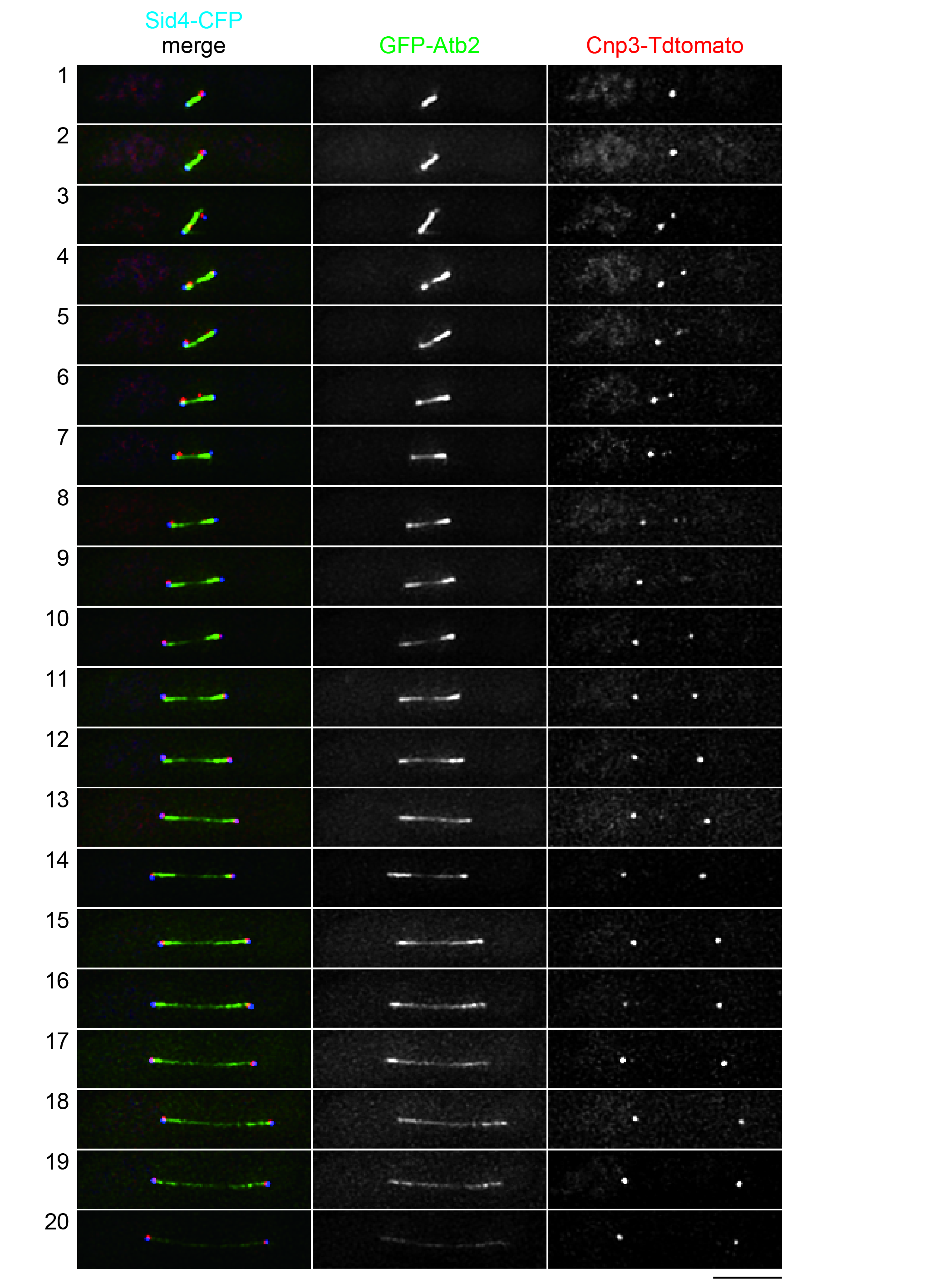

Supplement: Figure S4 — Sequential images of the kis1-1 cell of Figure 3D taken at 1-min intervals. (TIF) [file pone.0111905.s004.tif]

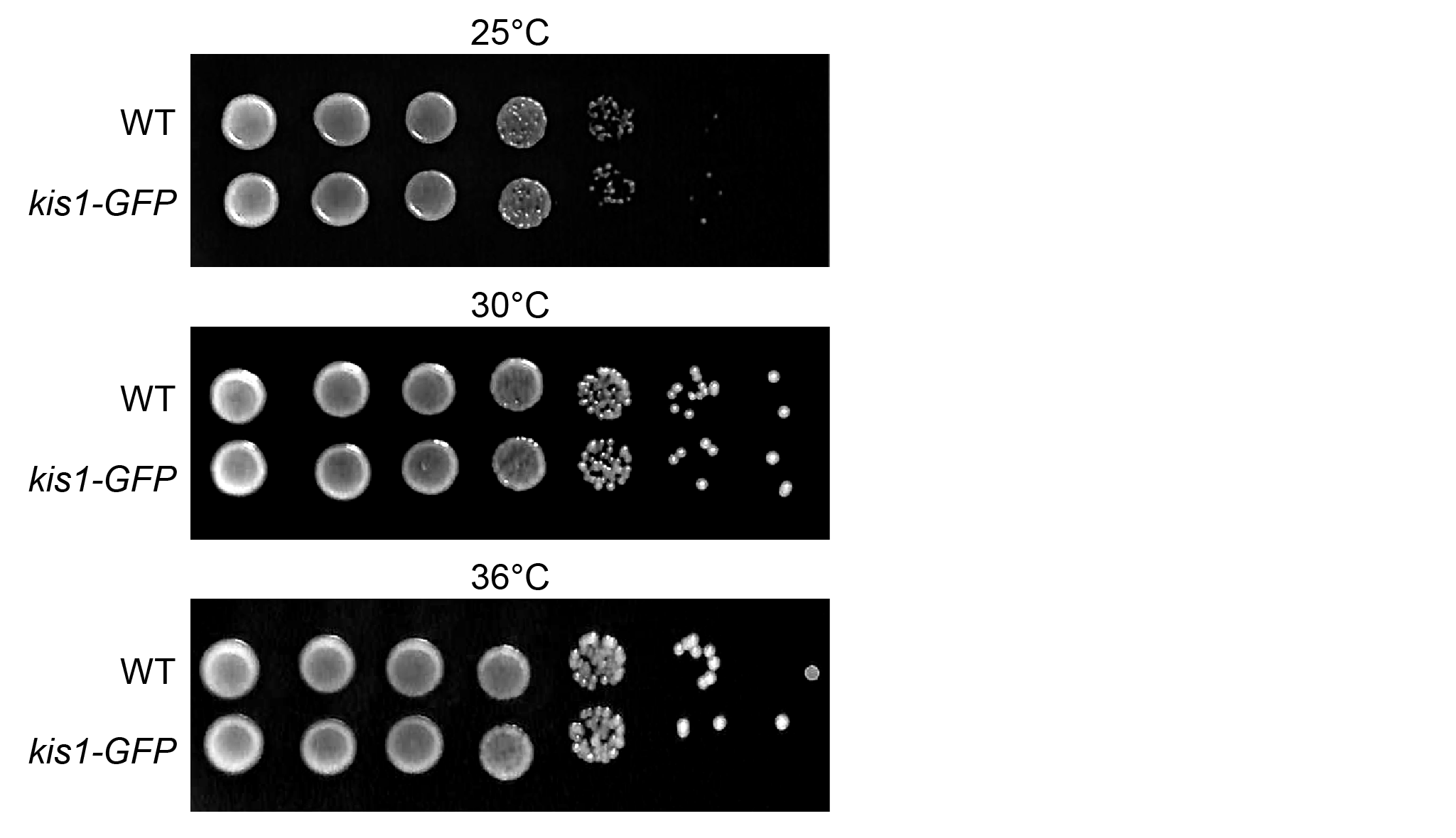

Supplement: Figure S5 — GFP-tagging of Kis1 does not affect cell proliferation. Ten-fold serial dilution assays of cells expressing GFP-tagged Kis1 (kis1-GFP) or non-tagged Kis1 (WT) were spotted on YE5S plates and incubated at 25, 30, or 36°C. (TIF) [file pone.0111905.s005.tif]

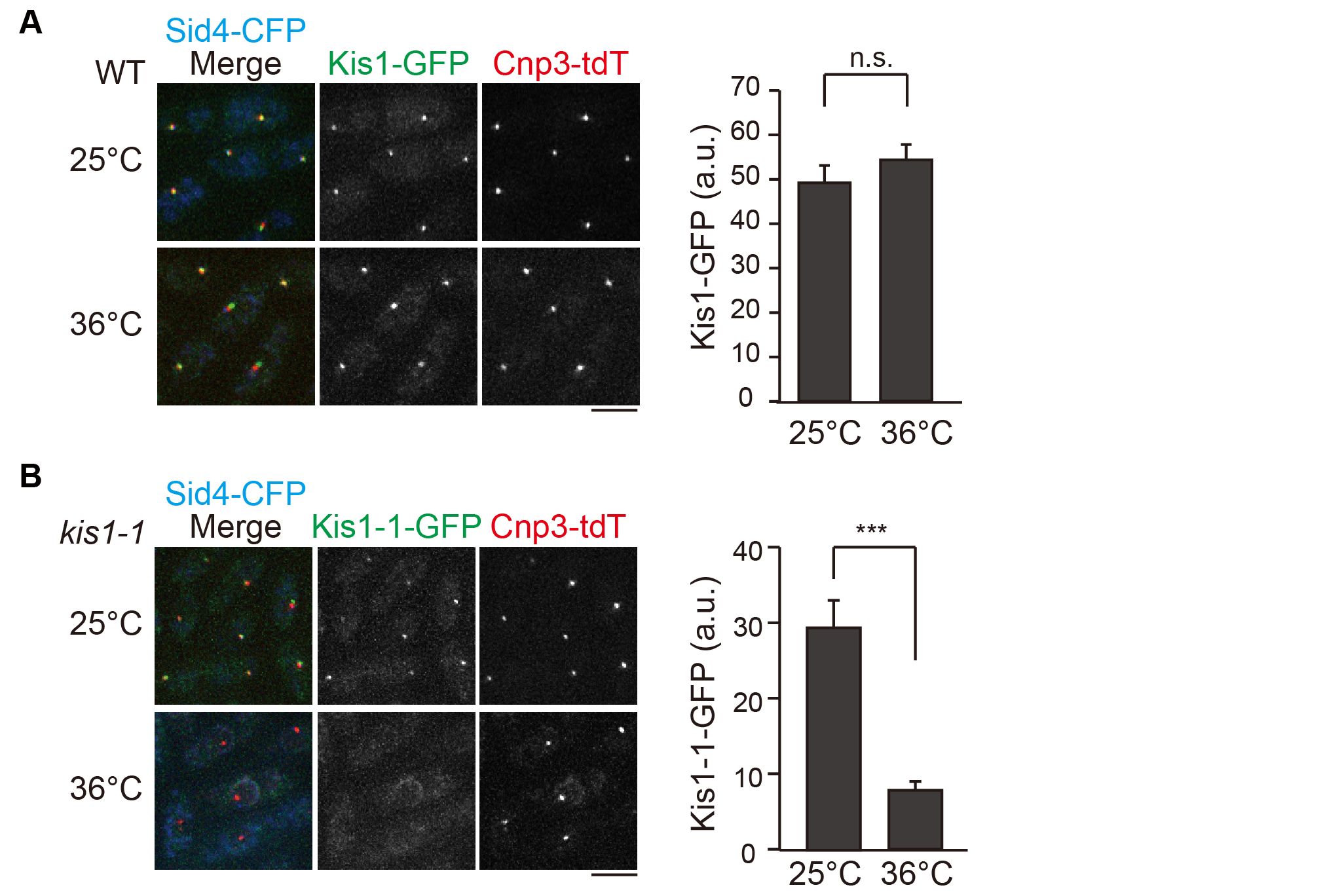

Supplement: Figure S6 — Kis1-1-GFP did not localize to kinetochores at 36°C. Cells expressing GFP-tagged Kis1 (WT, A) or GFP-tagged Kis1-1 (B) were visualized together with Sid4-CFP and Cnp3-tdTomato at 25 or 36°C (6 h). Left: Images for representative cells. Right: GFP fluorescence intensity at kinetochores in interphase cells observed under the indicated conditions. ***p<10−4 (Student's t-test); n.s., not significant (p>0.05), n≥10. (TIF) [file pone.0111905.s006.tif]

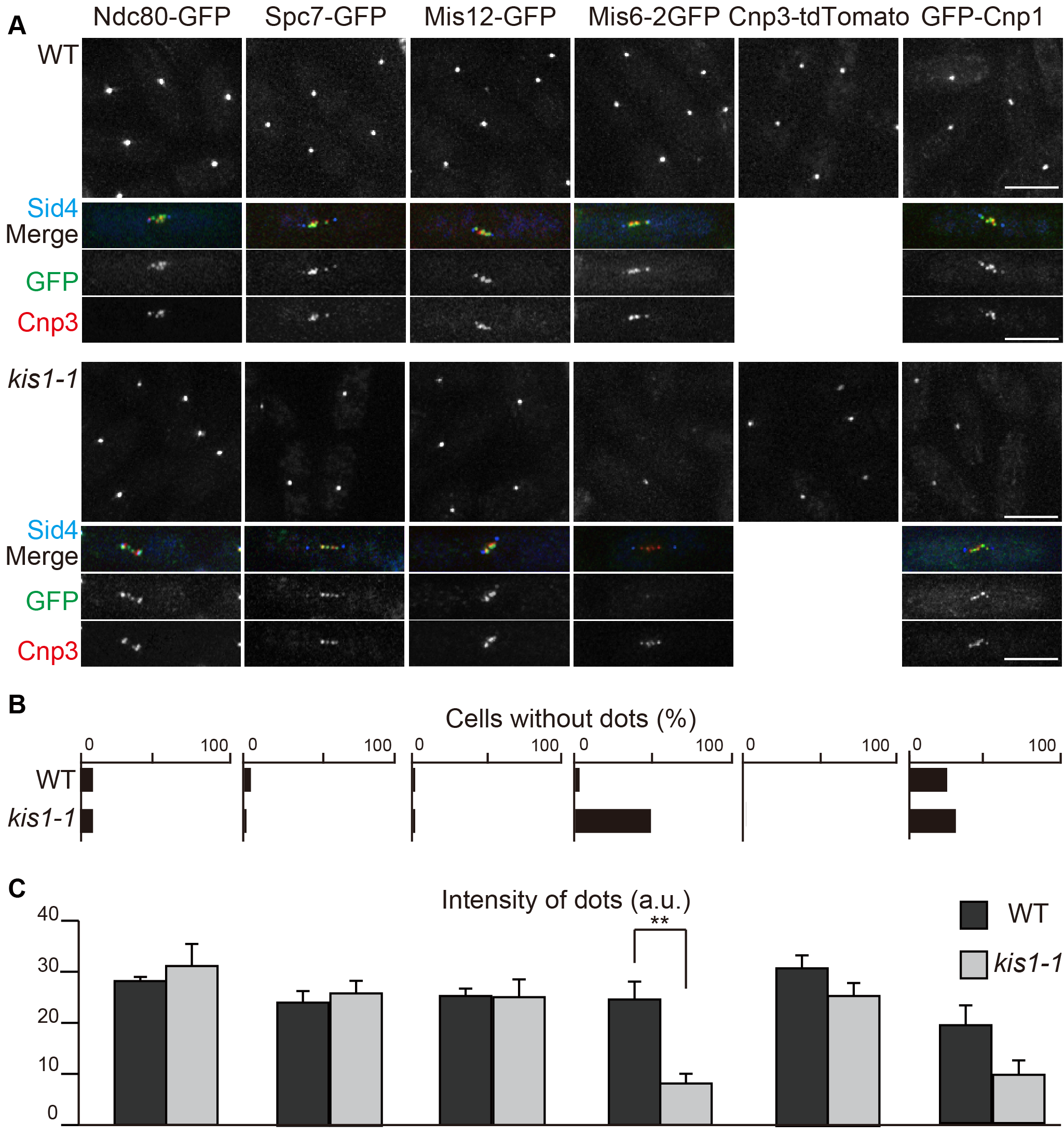

Supplement: Figure S7 — Localization of kinetochore proteins in WT or kis1-1 at 25°C. Cells were cultured at 25°C (the permissive temperature) and imaged. These data serve as controls for Figure 6C–E, in which cells were cultured at 36°C (the restrictive temperature). (A) Localization of the indicated kinetochore proteins observed in WT or kis1-1 cells. For mitotic cells, Sid4-CFP and Cnp3-tdTomato are also shown below. (B) Frequency of interphase cells without GFP localization at kinetochores for each kinetochore factor. n>50. (C) GFP or tdTomato fluorescence intensity of each kinetochore factor in WT (black) or kis1-1 (gray) cells. n≥10. ***p<10−4; ****p<10−6 (Student's t-test). Scale bars, 5 µm. (TIF) [file pone.0111905.s007.tif]

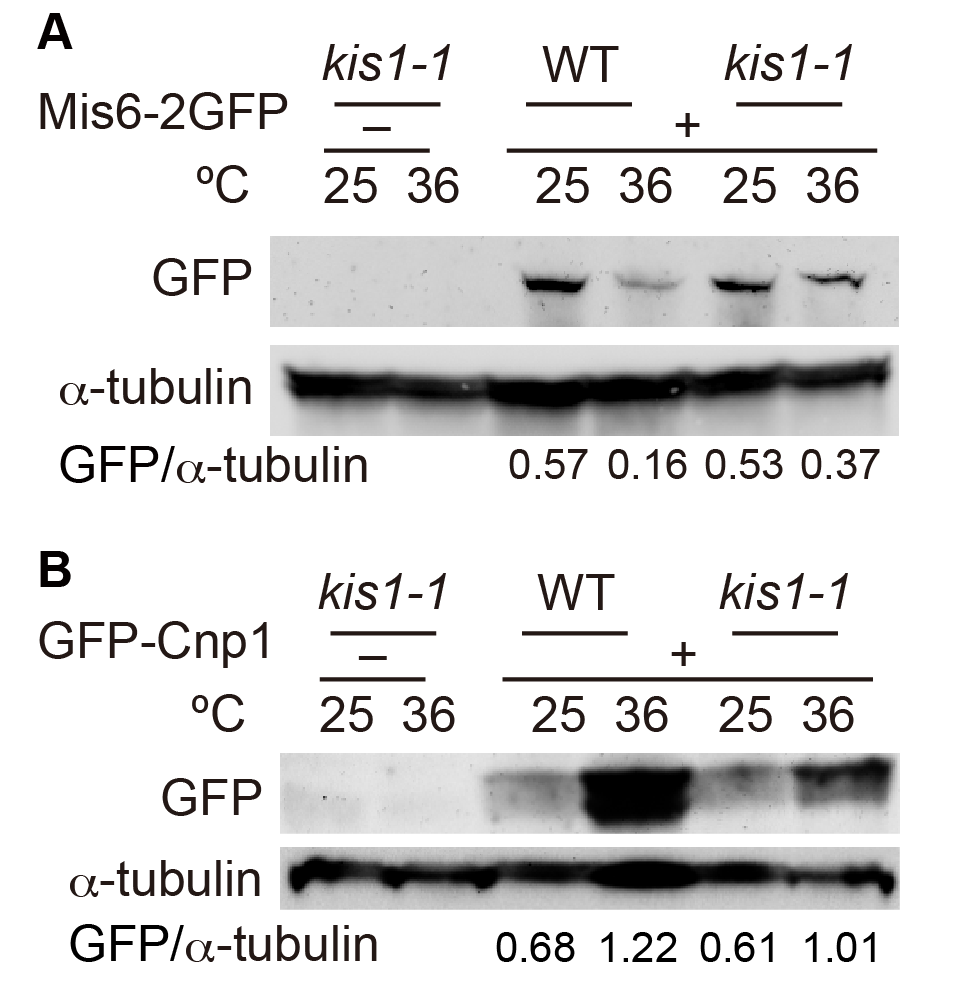

Supplement: Figure S8 — Amount of Mis6-2GFP and GFP-Cnp1 in WT or in kis1-1 . Cell extracts were prepared from WT or kis1-1 cells expressing Mis6-2GFP (A) or GFP-Cnp1 (B) cultured at 25 or 36°C (6 h). Immunoblotting was performed with anti-GFP and anti-α-tubulin. Cell extracts without Mis6-2GFP and GFP-Cnp1 are also shown as negative controls (–). A ratio value is shown for the Mis6-2GFP band intensity (A) or GFP-Cnp1 (B) intensity compared with that for α-tubulin. (TIF) [file pone.0111905.s008.tif]

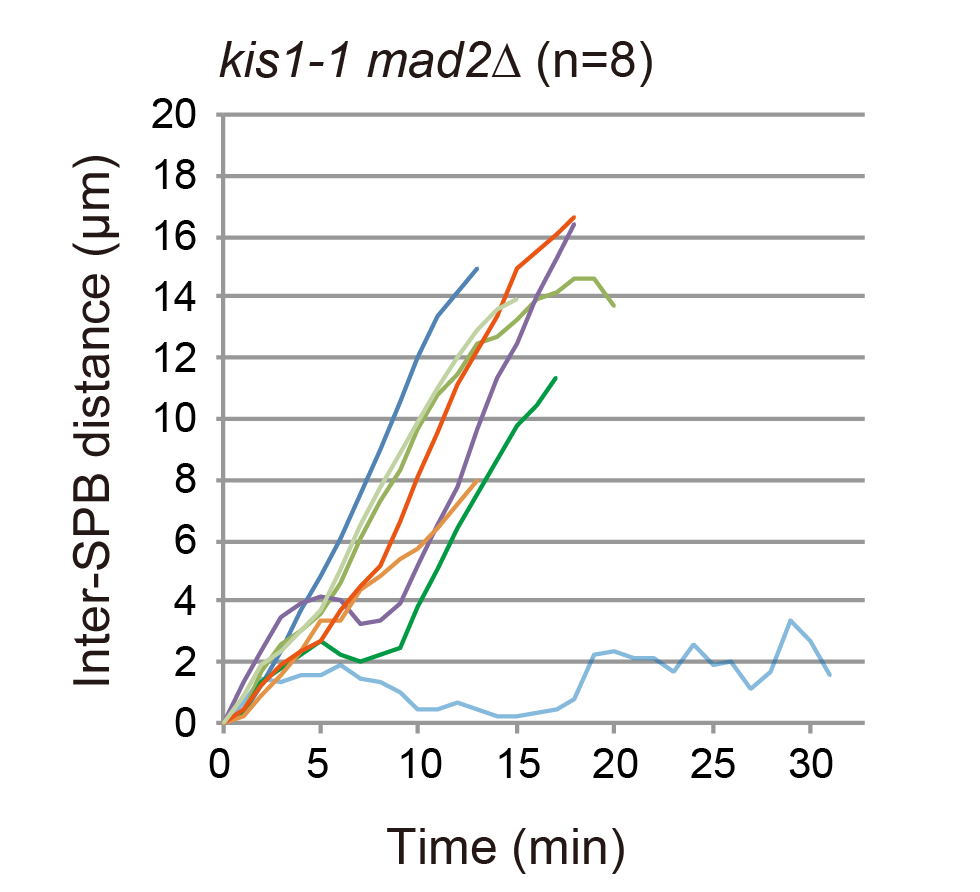

Supplement: Figure S9 — Kinetics of the inter-SPB distance in the kis1-1 mad2Δ double mutant. The kis1-1 mad2Δ double mutant cells expressing Sfi1-GFP (SPB) were grown at 25°C, followed by a temperature shift to 36°C for 6–9 h. Images were acquired every minute, and the inter-SPB distance was measured for each time point. n = 8. (TIF) [file pone.0111905.s009.tif]
