# Supplementary material for: The Kinetochore Protein Kis1/Eic1/Mis19 Ensures the Integrity of Mitotic Spindles through Maintenance of Kinetochore Factors Mis6/CENP-I and CENP-A
Source: PLoS One. 2014 Nov 6;9(11):e111905. doi: 10.1371/journal.pone.0111905 (PMC4222959; doi:10.1371/journal.pone.0111905)
Supplement: Table S1 — Fission yeast strains used in this study. (PDF) [file pone.0111905.s010.pdf]

Table S1. Fission yeast strains used in this study

Strain No.	Genotype	Figures Movies
KA2165	h^{-} *Z2-GFP-atb2-kan nup40-mCherry-hph sfi1-CFP-nat leu1 ura4 ade6-M210 CM3112sup3-5>>ade6-M216-bsd*	1A-C, S1
KA2193	h^{90} *mis6-302 Z2-GFP-atb2-kan nup40-mCherry-hph sfi1-CFP-nat leu1 ura4 ade6-M210 CM3112sup3-5>>ade6-M216-bsd*	1C
HH22	h^{+} *Z2-GFP-atb2-kan cnp3-tdTomato-hph sid4-CFP-nat his2 leu1 ura4 ade6-M216*	3A, B, M1
HH20	h^{+} *kis1-1 Z2-GFP-atb2-kan cnp3-tdTomato-hph sid4-CFP-nat his2 leu1 ura4 ade6-M216*	3A, D, E, S4, M2
HH231	h^{+} *sfi1-GFP-kan leu1 ura4*	3C
HH228	h^{-} *kis1-1 sfi1-GFP-kan leu1 ura4 ade6-M216*	3F
KRY11	h^{-} *kis1-1 sfi1-CFP-nat leu1 ura4 ade6-M210*	4B
KRY70	h^{+}/ h^{-} *kis1::kan/+ his2/+ leu1/leu1ura4/ura4 ade6-M216/ade6-M210*	4E
KRY128	h^{90} *cen2<<lacO-kan-ura4*$^{+}$ *his7*$^{+}$*<<(dis1pro)-GFP-lacI sfi1-CFP-nat his2 leu1 ura4 ade6-M216*	4F, G
KRY142	h^{-} *kis1-1 cen2<<lacO-kan-ura4*$^{+}$ *his7*$^{+}$*<<(dis1pro)-GFP-lacI sfi1-CFP-nat leu1 ura4 ade6-M216*	4F, G
KRY213	h^{+} *kis1-GFP-kan cnp3-tdTomato-hph sid4-CFP-nat his2 leu1 ura4 ade6-M216*	5A, C, 7D
KRY149	h^{90} *kis1-GFP-kan mis6-2mRFP-hph sfi1-CFP-nat leu1 ura4 ade6-M210*	5B,E
HH379	h^{-} *cdc25-22 kis1-1-GFP-kan*	5D
KRY202	h^{90} *cdc2-as-M17-bsd kis1-GFP-kan mis6-2mRFP-hph sfi1-CFP-nat leu1 ura4 ade6-M216*	5E
HH112	h^{90} *cut12-GFP-kan cnp3-tdTomato-hph leu1 ura4 ade6-M210*	6B
HH109	h^{+} *kis1-1 cut12-GFP-kan cnp3-tdTomato-hph his2 leu1 ura4 ade6-M216*	6B
HH102	h^{+} *pcp1-GFP-kan cnp3-tdTomato-hph his2 leu1 ura4 ade6-M216*	6B
HH96	h^{90} *kis1-1 pcp1-GFP-kan cnp3-tdTomato-hph leu1 ura4 ade6-M216*	6B
HH101	h^{+} *sfi1-GFP-kan cnp3-tdTomato-hph spo15-CFP-nat his2 leu1 ura4 ade6-M210*	6B
HH95	h^{+} *kis1-1 sfi1-GFP-kan cnp3-tdTomato-hph spo15-CFP-nat his2 leu1 ura4 ade6-M210*	6B
KRY209	h^{90} *ndc80-GFP-kan cnp3-tdTomato-hph sid4-CFP-nat leu1 ura4 ade6-M216*	6C-E, S7

HH74	h^{+} *kis1-1 ndc80-GFP-kan cnp3-tdTomato-hph sid4-CFP-nat his2 leu1 ura4 ade6-M210*	6C-E, S7
KRY212	h^{+} *spc7-GFP-kan cnp3-tdTomato-hph sid4-CFP-nat his2 leu1 ura4 ade6-M216*	6C-E, S7
HH77	h^{+} *kis1-1 spc7-GFP-kan cnp3-tdTomato-hph sid4-CFP-nat his2 leu1 ura4 ade6-M216*	6C-E, S7
KRY206	h^{90} *mis12-GFP-kan cnp3-tdTomato-hph sid4-CFP-nat leu1 ura4 ade6-M216*	6C-E, S7
HH73	h^{+} *kis1-1 mis12-GFP-kan cnp3-tdTomato-hph sid4-CFP-nat his2 leu1 ura4 ade6-M216*	6C-E, S7
KRY211	h^{+} *mis6-2GFP-kan cnp3-tdTomato-hph sid4-CFP-nat leu1 ura4 ade6-M216*	6C-E, 7A S7
HH78	h^{+} *kis1-1 mis6-2GFP-kan cnp3-tdTomato-hph sid4-CFP-nat leu1 ura4 ade6-M216*	6C-E, 7A S7
HH375	h^{-} *kis1-1 cnp3-tdTomato-hph sid4-CFP-nat leu1 ura4 ade6-M210 + pREP1-GFP-cnp1*	6C-E, S7
HH376	h^{-} *cnp3-tdTomato-hph sid4-CFP-nat leu1 ura4 ade6-M216 + pREP1-GFP-cnp1*	6C-E, S7
HH58	h^{-} *mis6-2GFP-kan leu1 ura4*	7B, S8A
HH81	h^{+} *kis1-1 mis6-2GFP-kan his2 leu1 ura4*	7B
HH3	h^{-} *kis1-GFP-kan leu1 ura4 ade6-M216*	7C, S5
HH147	h^{+} *mis6-302 kis1-GFP-kan cnp3-tdTomato-hph sid4-CFP-nat his2 leu1 ura4 ade6-M216*	7D
HH113	h^{-} *leu1 ura4 ade6-M216 + pREP1-GFP*	7E
HH136	h^{-} *kis1-1 leu1 ura4 ade6-M216 + pREP1-GFP*	7E
HH316	h^{-} *leu1 ura4 ade6-M216 + pREP1-mis6-GFP*	7E
HH368	h^{-} *kis1-1 leu1 ura4 ade6-M216 + pREP1-mis6-GFP*	7E
HH40	h^{90} *mis6-302 Z2-GFP-atb2-kan nup40-mCherry-hph sfi1-CFP-nat leu1 ura4 ade6-M210*	7G
KA2091	h^{-} *Z2-GFP-atb2-kan nup40-mCherry-hph sfi1-CFP-nat leu1 ura4 ade6-M210*	7G
KA2646	h^{+} *kis1-1 Z2-GFP-atb2-kan nup40-mCherry-hph sfi1-CFP-nat his2 leu1 ura4 ade6-M210*	7G
HH30	h^{+} *kis1-1 his2 leu1 ura4 ade6-M210*	8A
HH32	h^{-} *mis18-GFP-kan leu1 ura4 ade6-M216*	8A
HH51	h^{-} *mis16-GFP-kan leu1 ura4 ade6-M216*	8A, E
HH67	h^{+} *kis1-1 his2 leu1 ura4 ade6-M216*	8A

HH404	h^- *cnp3-tdTomato-hph sid4-CFP-nat leu1 ura4 ade6-M216 + pREP1-mis18-GFP*	8B
HH425	h^- *kis1-1 cnp3-tdTomato-hph sid4-CFP-nat leu1 ura4 ade6-M216 + pREP1-mis18-GFP*	8B
HH391	h^+ *kis1-GFP-kan cnp3-tdTomato-hph sid4-CFP-nat his2 leu1 ura4 ade6-M216*	8C, S6A
HH407	h^+ *mis18-262 kis1-GFP-kan cnp3-tdTomato-hph sid4-CFP-nat leu1 ura4 ade6-M216*	8C
HH416	h^- *mis16-53 kis1-GFP-kan cnp3-tdTomato-hph sid4-CFP-nat leu1 ura4*	8C
HH114	h^- *leu1 ura4 ade6-M216 + pREP1-GFP*	8D
HH398	h^- *cnp3-tdTomato-hph sid4-CFP-nat leu1 ura4 ade6-M210 + pREP1-mis16-GFP*	8D
HH137	h^- *kis1-1 leu1 ura4 ade6-M216 + pREP1-GFP*	8D
HH422	h^- *kis1-1 cnp3-tdTomato-hph sid4-CFP-nat leu1 ura4 ade6-M210 + pREP1-mis16-GFP*	8D
KA4859	h^- *kis1-myc-bsd mis16-GFP-kan leu1 ura4 ade6-M216*	8E
KA4874	h^- *kis1-myc-bsd mis18-GFP-kan leu1 ura4 ade6-M216*	8E
KA4858	h^- *kis1-myc-bsd leu1 ura4 ade6-M216*	8E
KRY208	h^- *dis1-GFP-kan cnp3-tdTomato-hph sid4-CFP-nat leu1 ura4 ade6-M216*	9A
HH83	h^+ *kis1-1 dis1-GFP-kan cnp3-tdTomato-hph sid4-CFP-nat his2 leu1 ura4 ade6-M216*	9A
HH222	h^{90} *mad2-GFP-kan cnp3-tdTomato-hph sid4-CFP-nat leu1 ura4 ade6-M216*	9B
HH388	h^{90} *kis1-1 mad2-GFP-kan cnp3-tdTomato-hph sid4-CFP-nat leu1 ura4 ade6-M216*	9B
JY745	h^- *leu1 ura4 ade6-M210*	9C
TM75	h^{90} *mad2::*$LEU2^+$ *leu1 ura4 ade6-M216*	9C
HH60	h^- *kis1-1 leu1 ura4 ade6-M216*	9C, S8A
HH245	h^{90} *kis1-1 mad2::*$LEU2^+$ *leu1 ura4 ade6-M216*	9C
HH363	h^- *leu1 ura4 ade6-M210*	S1
HH364	h^- *Z2-GFP-atb2-kan nup40-mCherry-hph sfi1-CFP-nat leu1 ura4 ade6-M210*	S1
HH365	h^- *leu1 ura4 ade6-M210 CM3112sup3-5>>ade6-M216-bsd*	S1
JY741	h^- *leu1 ura4 ade6-M216*	S5
HH8	h^+ *kis1-1-GFP-kan cnp3-tdTomato-hph sid4-CFP-nat his2 leu1 ura4 ade6-M216*	S6B
HH80	h^+ *kis1-1 mis6-2GFP-kan his2 leu1 ura4 ade6-M216*	S8A
HH357	h^- *kis1-1 leu1 ura4 ade6-M216 + pREP1*	S8B
HH117	h^- *leu1 ura4 ade6-M216 + pREP1-GFP-cnp1*	S8B

HH138	*h⁻ kis1-1 leu1 ura4 ade6-M216 + pREP1-GFP-cnp1*	S8B
HH314	*h⁻ kis1-1 mad2:: LEU2⁺ sfi1-GFP-kan leu1 ura4 ade6-M216*	S9

All strains originated in the present study, except for JV39, PN513, JY741, JY745 and TM75 (our stock).
