# Supplementary material for: The Kinetochore Protein Kis1/Eic1/Mis19 Ensures the Integrity of Mitotic Spindles through Maintenance of Kinetochore Factors Mis6/CENP-I and CENP-A
Source: PLoS One. 2014 Nov 6;9(11):e111905. doi: 10.1371/journal.pone.0111905 (PMC4222959; doi:10.1371/journal.pone.0111905)
Supplement: Table S2 — The number of mutants classified into one or two categories. In the screen, each mutant was classified into 1 or more of 12 categories according to phenotype. Category names are the same as shown in Figure 2, labeled as (A) to (L). Some mutants exhibited two distinct phenotypes and thus were assigned to both relevant categories. For example, 17 mutants belong to both (A) and (C) categories. The number of mutants classified into only one (“in one”) or in two categories (“in two”) is shown for each cell. Total numbers are also shown as a reference. (PDF) [file pone.0111905.s011.pdf]

Table S2. The number of mutants which were categorized in the screen

			in one	in two											
		Total		(A)	(B)	(C)	(D)	(E)	(F)	(G)	(H)	(I)	(J)	(K)	(L)
(A)	Monopolar spindles.	220	124		4	17	0	0	23	4	5	0	30	11	2
(B)	The middle region of the spindle was weak.	25	0	4		13	1	0	2	0	4	0	1	0	0
(C)	Accumulation of cells with the metaphase spindle.	113	44	17	13		11	0	3	2	3	0	15	5	0
(D)	The spindle was bent in anaphase.	50	31	0	1	11		0	2	0	1	0	1	3	0
(E)	Extremely short microtubules.	10	4	0	0	0	0		0	0	1	0	5	0	0
(F)	The number of microtubule bundles were smaller than in WT cells.	104	44	23	2	3	2	0		0	8	1	13	8	0
(G)	Microtubules were elongated and curvy at cell tips.	76	58	4	0	2	0	0	0		1	4	3	4	0
(H)	Microtubules were formed in the nucleus during interphase.	89	51	5	4	3	1	1	8	1		2	9	4	0
(I)	Microtubules were tethered around the cell tip.	25	14	0	0	0	0	0	1	4	2		3	1	0
(J)	The nuclear envelope was fragmented.	241	147	30	1	15	1	5	13	3	9	3		14	0
(K)	Cells showing more than two nuclei.	167	117	11	0	5	3	0	8	4	4	1	14		0
(L)	Multi-septated cells.	10	8	2	0	0	0	0	0	0	0	0	0	0	

One category: 642 colonies, Two category: 244 colonies
